# Far-infrared therapy promotes exercise capacity and glucose metabolism in mice by modulating microbiota homeostasis and activating AMPK

**DOI:** 10.1038/s41598-024-67220-5

**Published:** 2024-07-15

**Authors:** Shuo Li, Xiao-yao Miao, Jin-shui Zhang, Dong-dong Wei, Hua-jin Dong, Rui Xue, Jing-cao Li, Yang Zhang, Xiao-xing Feng, Jin Li, You-zhi Zhang

**Affiliations:** 1 State Key Laboratory of Toxicology and Medical Countermeasures, Beijing Key Laboratory of Neuropsychopharmacology, Beijing Institute of Pharmacology and Toxicology, Beijing, China; 2 Department of Pharmaceutical Science, Beijing Institute of Radiation Medicine, Beijing, China; 3 School of Medicine, Anhui University of Science and Technology, Huainan, 232001 China; 4 Grahope New Materials Technologies Inc., Shenzhen, 518063 China

**Keywords:** Graphene, Far-infrared, AMPK, Glucose metabolism, Gut-muscle axis, Physiology, Health care, Medical research, Molecular medicine

## Abstract

The benefits of physical exercise on human health make it desirable to identify new approaches that would mimic or potentiate the effects of exercise to treat metabolic diseases. However, whether far-infrared (FIR) hyperthermia therapy could be used as exercise mimetic to realize wide-ranging metabolic regulation, and its underling mechanisms remain unclear. Here, a specific far-infrared (FIR) rays generated from graphene-based hyperthermia devices might promote exercise capacity and metabolisms. The material characterization showed that the graphene synthesized by chemical vapour deposition (CVD) was different from carbon fiber, with single-layer structure and high electrothermal transform efficiency. The emission spectra generated by graphene-FIR device would maximize matching those adsorbed by tissues. Graphene-FIR enhanced both core and epidermal temperatures, leading to increased blood flow in the femoral muscle and the abdominal region. The combination of microbiomic and metabolomic analysis revealed that graphene-FIR modulates the metabolism of the gut-muscle axis. This modulation was characterized by an increased abundance of short-chain fatty acids (SCFA)-producing bacteria and AMP, while lactic acid levels decreased. Furthermore, the principal routes involved in glucose metabolism, such as glycolysis and gluconeogenesis, were found to be altered. Graphene-FIR managed to stimulate AMPK activity by activating GPR43, thus enhancing muscle glucose uptake. Furthermore, a microbiota disorder model also demonstrated that the graphene-FIR effectively restore the exercise endurance with enhanced p-AMPK and GLUT4. Our results provided convincing evidence that graphene-based FIR therapy promoted exercise capacity and glucose metabolism via AMPK in gut-muscle axis. These novel findings regarding the therapeutic effects of graphene-FIR suggested its potential utility as a mimetic agent in clinical management of metabolic disorders.

## Introduction

The human body benefits from exercise training, which enhances the cardiovascular system and regulates metabolism. Obesity, diabetes, cancer, and the burden of heart disease appear to be correlated with sedentary lifestyles^1^. According to reports, inactive individuals have shorter life expectancies than physically active individuals^2^. Many individuals and patients with these illnesses, however, are unable or unwilling to engage in physical activity. Metabolic modulators like PPAR agonist GW501516 and AMP-activated protein kinase (AMPK) agonist, termed "exercise mimetics", have been explored for their potential to emulate the favorable effects of aerobic exercise. These agents show promise in aiding the management of obesity and diabetes and in enhancing endurance, independent of physical training^3^.

According to a previous study, aldometanib, an AMPK agonist, increases the lifespan of mice^4^. However, due to abuse issues, there is significant discussion about the use of AMPK activators. AICAR, as an AMPK activator, is included in the prohibited list of substances by the World Anti-Doping Agency^5^. Additionally, a single pharmacological substance would not be adequate to simulate the complicated and extensive effects of physical activity because exercise has broad-ranging impacts on numerous types of cells, tissues, and organs.

The whole-body far-infrared ray (FIR) hyperthermia, also known as Waon therapy, has been employed in the management of diverse pathological conditions and symptomatic relief^6^. Previous studies demonstrated that FIR hyperthermia therapy has anti-ischemic condition, anti-inflammatory, anti-depression activity based on the biological activities of radiation^7,8^. Impressively, hyperthermia therapy contributes to the prevention and treatment of obesity through the activation of beige fat, mediated by the HSF1-A2B1 transcriptional axis^9^. It has also been reported that hyperthermia prevented bone loss by influencing the gut microbiota^10^. However, it is not clear whether far-infrared (FIR) hyperthermia therapy could be used as exercise mimetic to realize wide-ranging metabolic regulation.

FIR radiation (3–1000 µm) transmits energy solely as radiant heat that can reach up to 4 cm below the skin. In addition to resonating at cellular frequencies, FIR energy can induce rotational and vibrational motions in cellular molecules, particularly affecting water molecules^11^. The human skin can efficiently absorb the efficient infrared radiation produced by FIR emitters, which resonates with the human body and accelerates metabolism and blood flow^12^. Non-absorbed infrared radiation may build up on the body's surface and cause skin abrasion. The FIR devices receive energy from the human body, acting as "absorbers" and keeping their temperature high enough to re-emit the FIR back to the body^6^.

The efficacy of FIR hyperthermia has been augmented with the advent of improved methods for generating pure FIR emissions. However, FIR hyperthermia equipment with high resonance effect and electrical stability is absent in practical applications. Moreover, achieving an optimal balance between therapeutic efficiency and minimizing adverse effects poses a significant challenge. Resonance adsorption would increase adsorption efficiency and reduce side effects in living tissue, making it desirable to have a safe FIR generating source whose emission spectra match those adsorbed by tissues. Previous studies have shown that the blackbody limit in the far-field may be eluded by the radiative heat transfer rate at nanogaps^13–15^. Carbon-based nanomaterials, such as carbon nanotube and graphene, have atomically restricted lattice structures and can support a significantly higher current density than traditional metals, making them particularly potential thermal emitters^16,17^. Graphene film has a remarkable FIR emissivity over 90%, a large portion of electronic energy can be converted into infrared radiation when a bias voltage is applied through the graphene layer. The infrared spectrum characteristics of graphene do not change with radiation temperature^18^. However, the implications of graphene-FIR hyperthermia on enhancing physical activity and maintaining metabolic equilibrium as an exercise mimetic warrant further investigation.

It is now widely documented that the gut microbiota plays an important role in regulating host metabolism^19^. Recent research has demonstrated that the gut microbiota has a variety of effects on skeletal muscle bioenergetics. For example, micronutrients and metabolites derived from the gut microbiota affect energy homeostasis in muscle^20^. Probiotic supplementation in mice decreases muscular atrophy but increases skeletal muscle strength^21,22^. A current study investigates the induction of microbiome depletion in mice through the administration of a broad-spectrum antibiotic mixture (ABX) to explore the role of gut microbiota. Microbiota depletion in mice exhibit decreases in skeletal muscle mass, strength and mitochondrial function^20^. The notion of "gut-muscle axis" was proposed to describe how gut microbes influence muscle function and endurance performance^23^. It was reported that accelerated nitrogen recycling via gut symbionts may benefit for muscle preservation in humans^24^. The richness abundances of *Veillonella* and *Lactobacillus salivarius* in microbiomes could enhance exercise performance and decrease fatigue^25,26^. The skeletal muscle responds to endurance exercise by activating AMPK and remodeling metabolism. Narkar et al. had an intriguing reported that activating AMPK could mimic the benefits of exercise without training^27^. Previous studies suggested that AMPK play a key role in the regulation of skeletal muscle energy metabolism promoting glucose uptake and fatty acid oxidation^28^.

In the present study, we have demonstrated that graphene-FIR therapy different from carbon fiber-FIR, exhibiting enhanced electrothermal stability and a more pronounced resonance effect in FIR hyperthermia treatment. We found that graphene-FIR hyperthermia could promote exercise capacity and glucose metabolism via AMPK. We further providing a mechanistic understanding of the role of a gut-muscle interaction in hyperthermia therapy.

## Materials and methods

### Animals

All the animal experiment protocols were approved by the Committee on the Ethics of Animal Experiments of Academy of Military Medical Sciences and reported in accordance with the ARRIVE guidelines. Approval number: IACUC-DWZX-2022–632. We confirmed that all experiments were performed in accordance with relevant guidelines and regulations. The mice were euthanized by carbon dioxide. The C57BL/6 J male mice (Sibeifu, Beijing, China) utilized in the experiments were 8–14 weeks old. They were subjected to a 12-h light/12-h dark cycle and were kept in a temperature and humidity-controlled environment (24 ± 2 °C, 55 ± 5% relative humidity). The grouping information of animal experiments were provided in the Supporting Information Table 1. ABX-mediated microbiota perturbations model was established according to previously protocols (details are provided in Supplementary data).

### Materials

The details of materials are provided in Supplementary Data (including manufacturers and product numbers).

### Infrared spectrum analysis

The details of FIR spectrum detection and analysis can be found in Supplementary data.

### FIR irradiation and exposure

The mice were exposed to FIR radiation in a rectangular plastic chamber (40 cm × 25 cm × 20 cm). Each chamber contains 16 FIR films with series connection, the single films resistance value is 27 Ω. There are four films above, on the left, and on the right of the chamber separately, and two films in front and behind. The FIR films were installed in the surface of chamber and powered by an independent variable voltage power. During the FIR irradiation experiment, the mice were placed in the center of chamber and allow to free move. Except for the feet, the entire body of a mouse can receive FIR radiation. The temperature was approximately (24.0 ± 1) °C in the control cages and (30.0 ± 1) °C in the FIR chamber. The experiment room temperature was controlled at 25 °C. The input power of the graphene-based device and carbon fiber-based device were all set at 30 W. The animals were radiated for two consecutive weeks, 40 min daily.

### Exercise endurance test

Exercise endurance was determined using a treadmill (Sansbio, Jiangsu, China) running tests as previously described with minor modified^29^. The low and high-intensity exercise test were defined by setting different running time. The details of experimental can be found in Supplementary data.

### Locomotor activity

To examine the effect of FIR on the locomotor activity, 30 min or 24 h after the last FIR radiation (day 14), locomotor activity was examined in a 15 min monitoring period. The movement tracking was recording by using an automated video tracking system, as described previously. Eight test boxes (40 cm × 40 cm × 30 cm high) were operated simultaneously by a compatible computer.

### Core and surface body temperature

Body temperature of mice was monitored using Anipill loggers (BodyCap, Paris, France) to get temperature data from animals after Anipill transmitter implantation. Isoflurane was used to sedate mice before surgery. After inserting the Anipill logger, the skin was stitched using surgical suture. Prior to the studies, the animals were returned to the cage for four days of rest.

The surface temperature of subjects (mice and FIR film) was tested by the infrared imaging. IR imaging tested in a room with a steady temperature and humidity. Thermographic camera (UTi320E, China) was recorded temperature in mice or FIR film.

### Blood flow measurements/laser speckle contrast imaging (LSCI)

To prevent the carryover effects of differing acclimation temperatures, all mice were placed at 25 °C for 1 h before to research. The mice were placed in an RFLSI Pro (RWD life sciences) and kept at 1.2% isoflurane. A CCD camera detects blood flow and then captures the image. Regions of interest (ROIs) were chosen and assess speckle contrast.

### Western blot analysis

As previously reported, SDS-PAGE electrophoresis was used to separate an identical amount of protein, which was then transferred to a nitrocellulose membrane. 5% skim milk was used to block membranes for two hours. To maximize efficiency in isolating the target proteins, the strips will be cut post-blocking in alignment with the protein ladder. After primary antibody incubation for 12 h at 4 °C, secondary antibody was incubated for 2 h. Using a gel imaging analyzer (AlphaImager, USA), the optical density of the appropriate immunoreactive band was measured.

### AMPK, AMP and ATP assay

The fresh muscle samples were homogenized. The supernatant was incubated with the appropriate reaction solution and substrate solution in accordance with the kit's instructions. A luciferase reaction was used to measure the levels of AMPK, AMP, and ATP using a microplate reader (Envision 2104). A standard curve was used to connect the luminescence signal to the concentrations of AMPK, AMP, or ATP.

### Fluorescence imaging analysis

In vivo fluorescence imaging was detected and quantified by AniView600 imaging system (Guangzhou Biolight Biotechnology Co., Ltd). To reduce autofluorescence, animals were fasted for 12 h and hair was removed from joints. Animals underwent caudal vein injection solution of Cy5.5-glucose (2.5 mg/kg). As recommended by the system for image acquisition, the excitation and emission filters were set at 675 nm/Cy5.5 and 740 nm/Cy5.5, respectively. Animals were scanned before injection and at 3 h after injection. We calculated average Cy5.5-glucose signal in skeletal muscle.

### Metabolomics and microbiome analysis

The details of experimental protocols and analysis methods were provided in Supplementary data.

### Statistical analysis

The results were presented as the mean ± SEM. For comparisons involving three or more groups, one-way ANOVA was employed, followed by the Bonferroni test to determine significance. Body temperature data were statistically analyzed using the Holm-Sidak method. The correlation between metabolites and running distance was assessed using Pearson’s correlation test. *P* < 0.05 was regarded as statistically significant in each case. GraphPad Prism 8.01 was used to conduct statistical analysis.

## Results

### The emission spectrum of FIR rays by graphene-based device

**Figure 1:**
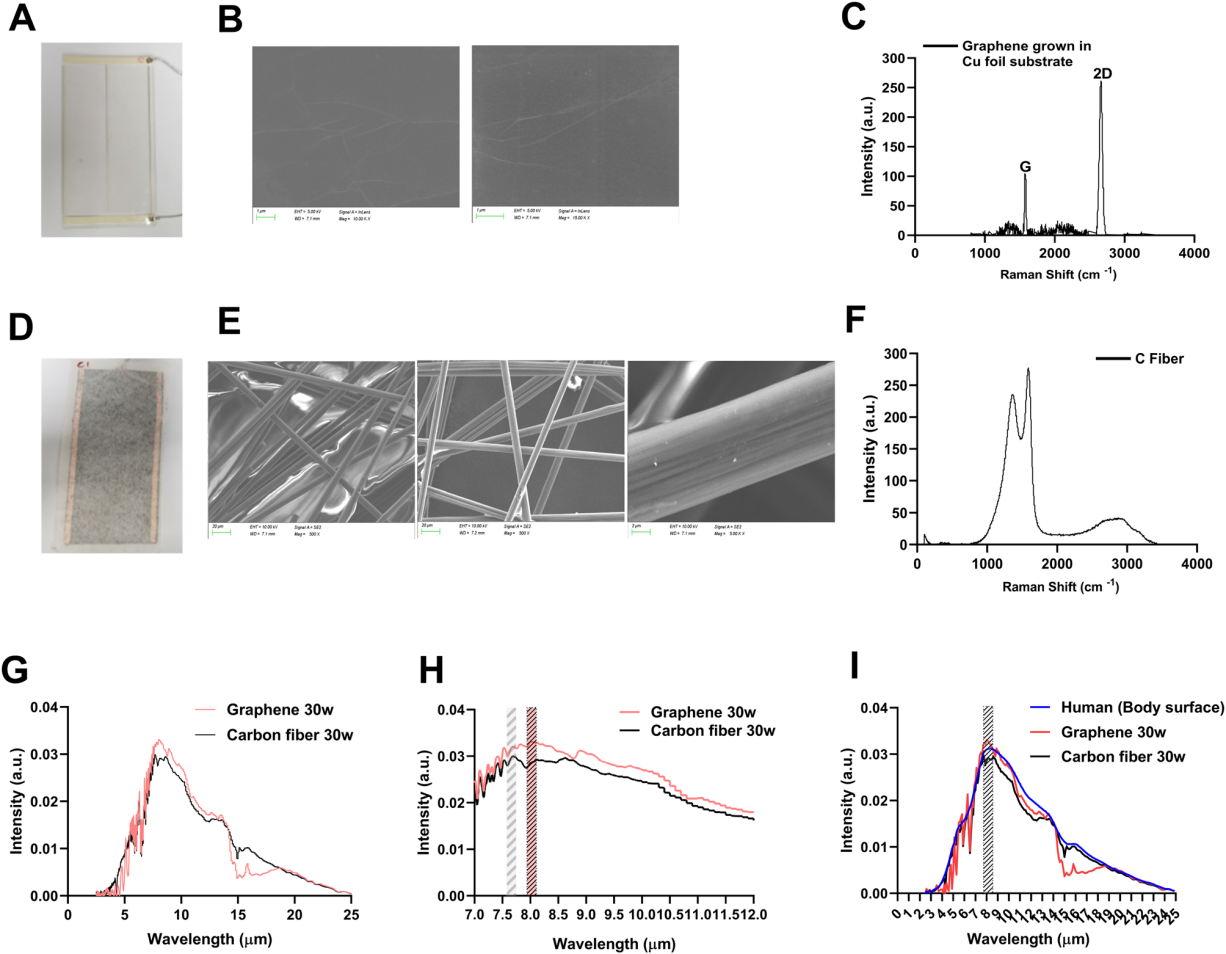
Specific FIR rays generated by graphene-based devices. (**A**) Photograph of a graphene film. (**B**) SEM image of graphene film. (**C**) Raman spectrum of the graphene FIR. (**D**) Photograph of a carbon fiber film. (**E**) SEM image of carbon fiber film. (**F**) Raman spectrum of the carbon fiber FIR. (**G**) FIR emission spectra of graphene and carbon fiber (graphene: 30 W, carbon fiber: 30 W). (**H**) The FIR emission peak of the graphene was approximately 8 µm, the carbon fiber was approximately 7.5 µm. (**I**) The absorption peak of the human body surface was approximately 8.0 µm.

First, we identified the material characterization of graphene film and carbon fiber. The single-layer graphene was grown using CVD on a copper foil and to a polyethylene terephthalate (PET) membrane (Fig. 1A), and the carbon fiber-based device was commercially available (Fig. 1D). The SEM images showing the morphology of graphene film and carbon fiber were different. The graphene film was two-dimensional membrane (Fig. 1B), and the carbon fiber was existed in the fibrous form of graphitized structure (Fig. 1E). Raman spectroscopy verified the single-layer nature of the graphene film (Fig. 1C), and the multi-layer nature of the carbon fiber (Fig. 1F). In the electrothermal characteristic detection, compared to the carbon fiber-based device, the power of the graphene-based device was more stable, and the heating rate is faster when under the same power condition (Fig. S1, Supporting Information).

The emission peak of the single-layer graphene device was different from carbon fiber device in the same input powers (Fig. 1G–H). We also assessed the human body's absorption spectra (Fig. 1I). The emission peak position of the graphene device (8.0 µm) coincided with the characteristic absorption peaks of the human body surface, which were around 8.0 µm. For the same power input, the carbon-fiber devices' emission peak wavelength was lower than 7.7 µm (Fig. 1H). These data showed that the FIR generated by graphene may have more efficiently resonated with epidermal molecules.

### Graphene-FIR hyperthermia increased exercise endurance in mice

**Figure 2:**
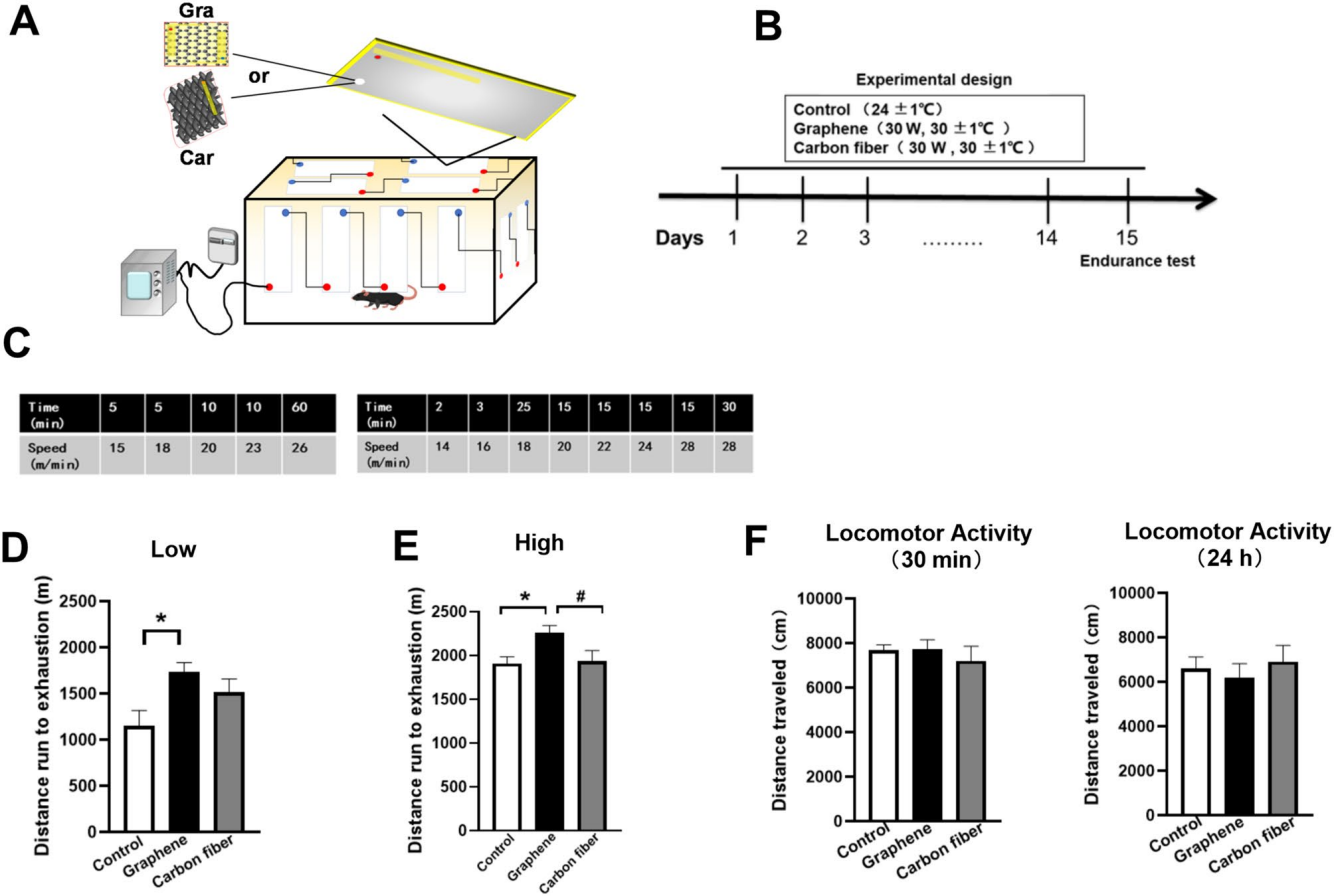
Graphene-FIR hyperthermia increased exercise endurance in mice. (**A**) Diagram of the generation of FIR rays by graphene-based and carbon fiber-based devices. (**B**) Experimental procedure. Mice received 40 min/day FIR radiation for 14 days. The endurance test was conducted on day 15. (**C**) Schematic depiction of the different treadmill exercise protocols. (**D**–**E**) Running distance on the treadmill for low and high exercise protocols. Distance until exhaustion on the treadmill after FIR irradiation. (**F**) The distance of locomotor activity 1 h or 24 h after the last FIR radiation. Data are expressed as mean ± SEM; n = 8 in each group, *p < 0.05 compared to Control group, ^#^*p* < 0.05 compared to Carbon fiber group, one-way ANOVA followed by Bonferroni test.

The diagram of FIR devices by graphene or carbon fiber and the experimental design were shown in Fig. 2A,B. Firstly, the body weight, food intake and water intake of mice were not changed by graphene or carbon fiber-FIR hyperthermia (Fig. S2A–C, Supporting Information). We examined the difference in exercise capacity of naive mice between control and FIR treatment groups. We tested the treadmill performance of mice under 2 different protocols: low and high intensities that generally correspond to 50% and 100% of their maximal aerobic capacity, respectively (Fig. 2C). In low-intensity exercise protocol, the graphene group showed greater running endurance than control mice, while the running distance of carbon fiber group was slightly increased but with no significant difference (Fig. 2D). As shown in high-intensity protocol (Fig. 2E), the graphene group also performed substantially better than the control group, and the running distance of graphene group was obviously higher than the carbon fiber group (*p* < 0.05). Neither control nor FIR-exposed animals showed any significant in spontaneous locomotor activity test after 30 min or 24 h of last FIR-hyperthermia, indicating that FIR-hyperthermia will not induce locomotor patterns parallel those of heat stress or psychomotor stimulant (Fig. 2F).

### Graphene-FIR hyperthermia improved body temperature and blood flow in mice

**Figure 3:**
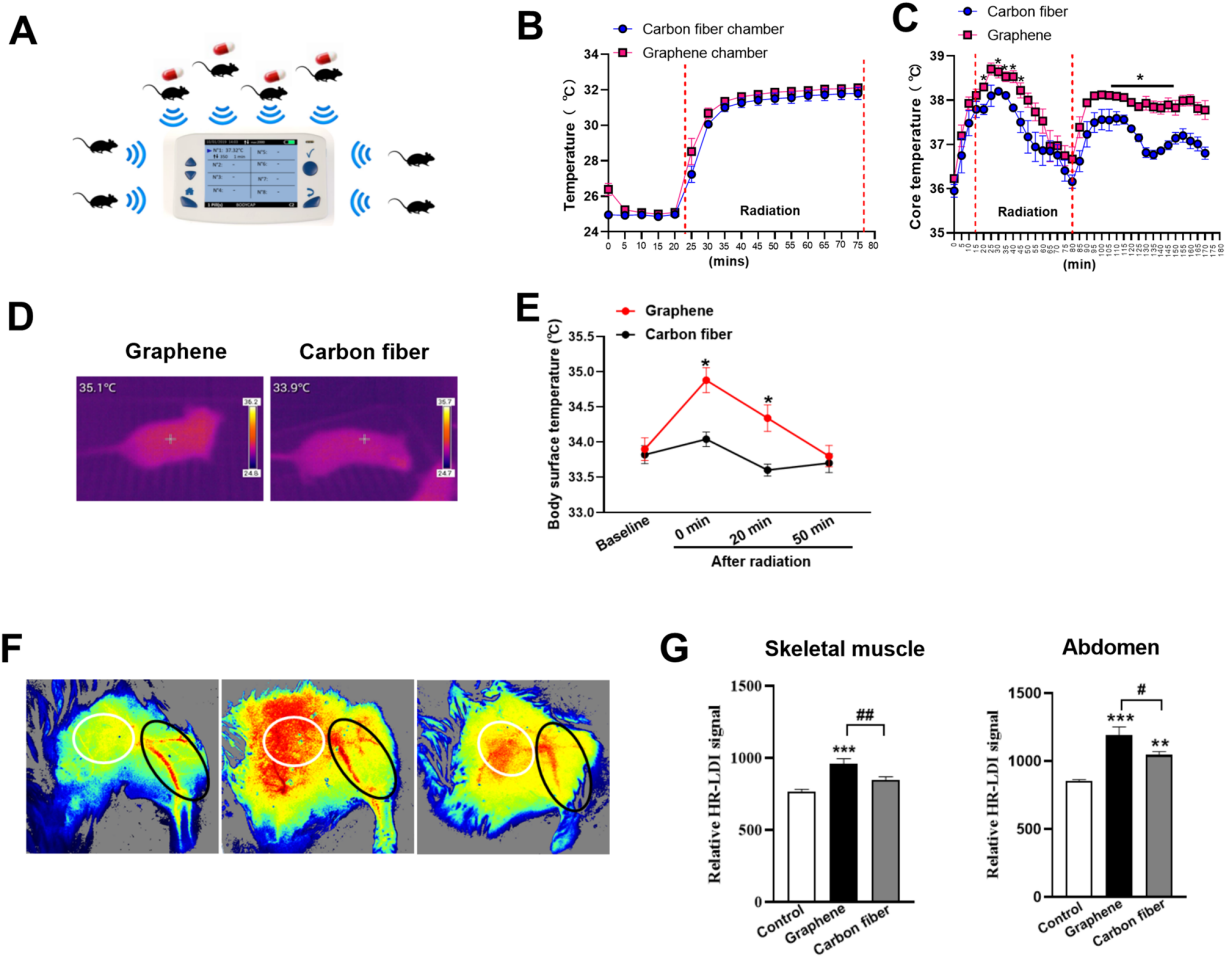
Graphene-FIR hyperthermia improved body temperature and blood flow in mice. (**A**) Body capsules and temperature monitor. (**B**) The ambient temperature in FIR chamber before and after radiation. (**C**) The core temperature of mice before and after radiation. (**D**) The representative infrared thermal images after FIR hyperthermia. (**E**) The body surface temperature of mice before and after radiation. (**F**) The representative blood flow images. (**G**) The quantitation of blood perfusion in skeletal muscle and abdomen. Data are expressed as mean ± SEM; n = 8 in each group, *p < 0.05 compared to Carbon fiber group, Tukey's multiple comparisons test, **p < 0.01 and ***p < 0.01 compared to Control group, ^#^*p* < 0.05 and ^##^*p* < 0.01 compared to Carbon fiber group, one-way ANOVA followed by Bonferroni test.

In present study, as showed similar spectral peaks in graphene-FIR and skin surface, whether FIR energy of graphene can improve body temperature more effectively. To test this hypothesis, core temperature of mice was recorded by using body capsules (Fig. 3A). The comparison of the data obtained from the two FIR chambers was firstly performed during FIR hyperthermia, there was no significant difference between graphene group and carbon fiber group (Fig. 3B). We then analyzed the core temperature of mice in different groups. During the FIR hyperthermia, core temperature of the graphene group exhibited significant rises compared to the carbon fiber group (Fig. 3C). In addition, temperature profiles of the graphene group exhibited similar significant rises near 38 °C at several time points immediately after the FIR hyperthermia. Thermographic images showed that the surface temperature in graphene group was significantly higher than carbon fiber group after FIR hyperthermia (Fig. 3D). We also observed the high level of the epidermal temperature will resist nearly 50 min, which consisted with the core temperature (Fig. 3E). Notably, our findings indicated that serum NE levels remained unchanged in mice following FIR hyperthermia (Fig. S3, Supporting Information), thereby ruling out the possibility of sympathetic activation due to heat stress and increased thermogenesis.

Using the RFLSI system, we further detected the blood flow in skeletal muscle and abdomen (Fig. 3F). Following continuous FIR hyperthermia, the blood flow was measured without acute hyperthermia. As shown in Fig. 3G, the HR-LDI signal increased significantly throughout the abdomen region in both graphene and carbon fiber groups. And the blood flow of graphene-FIR mice was higher than carbon fiber mice. We also found that the femoral muscle blood flow in graphene-FIR group was significantly higher than that of carbon fiber group. Taken together, these data suggested that graphene-FIR may promoted physiological changes in a metabolic regulation fashion.

### Graphene-FIR hyperthermia altered the structure of the gut microbiome

**Figure 4:**
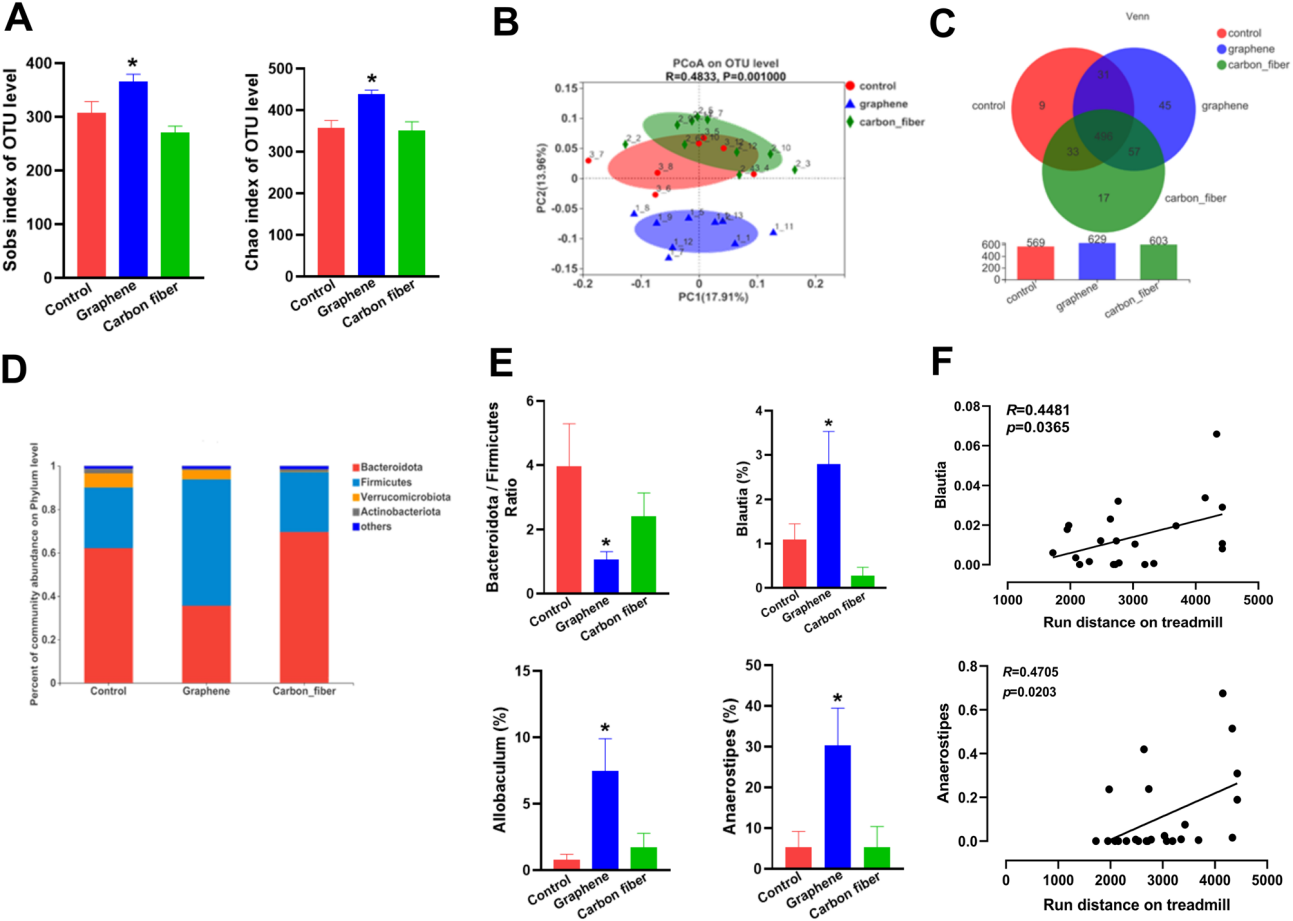
Graphene-FIR hyperthermia altered the structure of the gut microbiome. (**A**) α-diversity (Sobs and Chao) comparison of the gut microbiota. (**B**) Unweighted UniFrac distances plotted using principal component analysis (PCoA) and the relative abundance of OTUs. (**C**) Venn diagram analysis of OTU compositions in different groups. (**D**) Relative gut microbiota abundance at the phylum level in the different groups. (**E**) Relative abundance rates of *Bacteroidetes/Firmicutes*, *Blautia*, *Allobaculum* and *Anaerostipes* in control, graphene and carbon fiber mice. (**F**) Correlation analysis: regression plot between the abundance of *Blautia* or *Anaerostipes* and running distance. Data are expressed as mean ± SEM; n = 6 in each group, *p < 0.05 compared to Control group, one-way ANOVA followed by Bonferroni test.

Recent evidence suggested an interaction between the gut microbiota and exercise endurance. To investigate whether graphene-FIR hyperthermia can alter the microbiota composition, we performed 16S ribosomal DNA analysis of microbiota in colon after the endurance test. Diversity analyses reveal that there are variations between the graphene and control groups in the α- and β-diversities of the gut flora (Fig. 4A,B). The Sobs and Chao index of graphene group were significantly higher than these of control and carbon fiber group. The PCoA plot (Fig. 4B) shows that at baseline, the graphene group and control group were separated along the PC2 axis, however, the carbon fiber group and control group were converged to the same position. Venn diagram based on genera (Fig. 4C). The three groups have 496 shared genera, with 45 unique genera in the graphene group, 17 unique genera in the carbon fiber group and 9 unique genera in the control group. These findings showed that the community diversity of gut microbiota was increased by the graphene treatment.

In Fig. 4D, *Bacteroidetes* and *Firmicutes* dominated the gut microbiota composition at the phylum level, contributing to 62.2 and 28% of the gut microbiota in the control group, 35.6 and 58.3% in the graphene group, 69.7 and 27.5% in the carbon fiber group, respectively. The *Bacteroidetes*/*Firmicutes* (B/F) ratio was significantly reduced by graphene-FIR (Fig. 4E). We further studied the group differences at the genus level. Compared with the control group, SCFAs-producing bacteria including *Anaerostipes* (belonging to the butyrate producing bacteria) *Allobaculum* and *Blautia*, were induced by graphene-FIR hyperthermia in mice (Fig. 4E). They are genus of anaerobic bacteria with probiotic characteristics. We further analyzed the relationship between the gut microbiota and motor functions using pearson’s correlation (Fig. 4F). We found that the running distance of mice was significantly positively correlated with *Anaerostipes* (R = 0.4705, *P* = 0.0203), *Blautia* (R = 0.4481, *P* = 0.0365), respectively.

**Figure 5:**
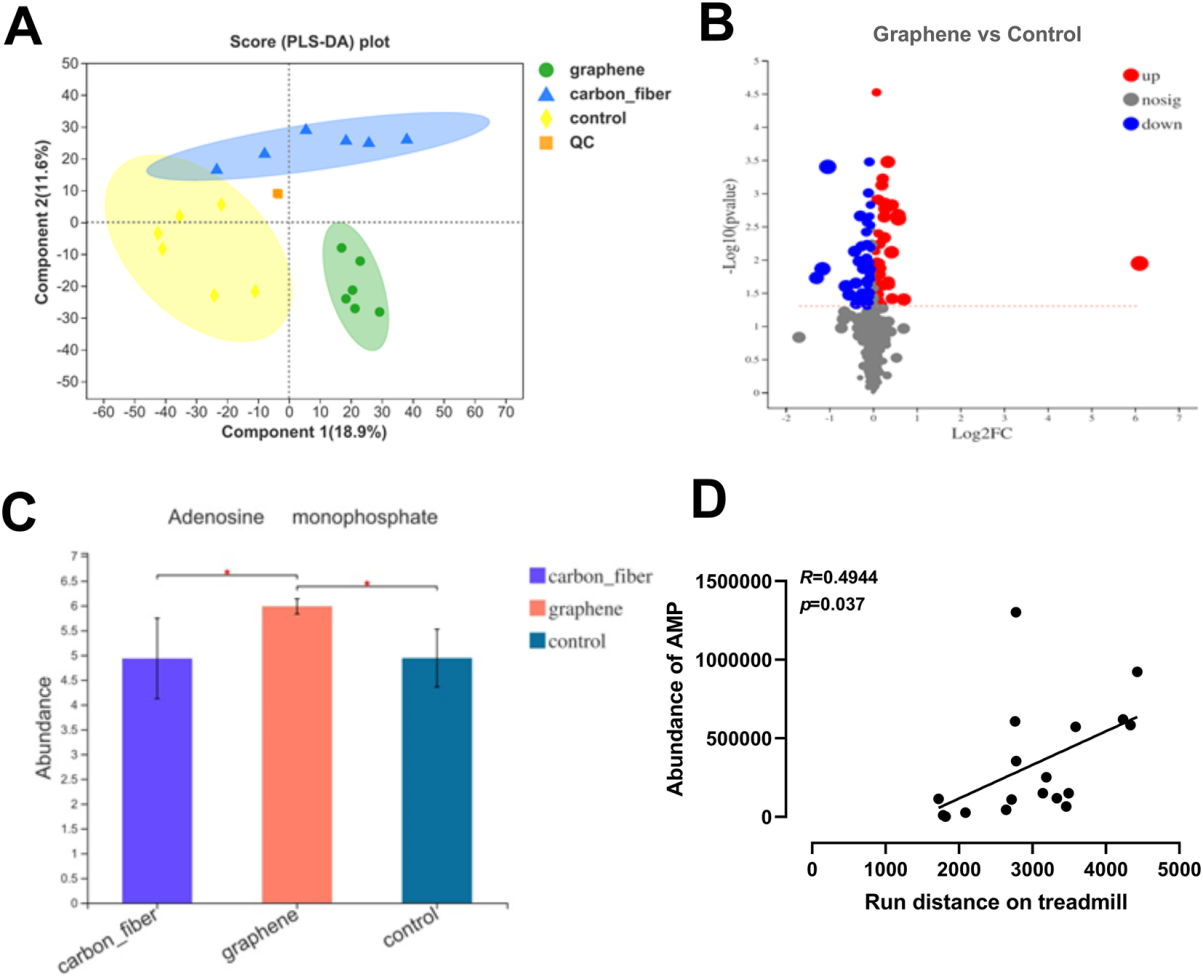
Graphene-FIR hyperthermia changes the metabolomic profile of the gut microbiome. (**A**) Partial least squares-discriminant analysis (PLS-DA). (**B**) A volcano plot of the metabolites. (**C**) Relative abundance rates of AMP in control, graphene and carbon fiber group. (**D**) Correlation analysis between the abundance of AMP and running distance. n = 6 in each group, *p < 0.05, **p < 0.01 and ***p < 0.001 compared to Con group or carbon fiber group, one-way ANOVA followed by Bonferroni test.

Fecal metabolic profiles of FIR treated group and control group were acquired by LC–MS. As shown in PLS-DA plots (Fig. 5A), a clear separation between the graphene-FIR group and other groups was found, suggesting that graphene-FIR hyperthermia led to significant biochemical changes. In volcano plot of the metabolites, x-axis denoted the fold changes of the metabolites in different groups and y-axis denoted the significance of the changes for the metabolites (Fig. 5B). The variation tendencies of metabolites were described by a heat map in Fig. S4A. 18 metabolites were significantly decreased and 32 were significantly increased in graphene-FIR mice compared with controls. Interestingly, the abundance of adenosine monophosphate (AMP) in graphene group was significantly higher than control and carbon fiber group (Fig. 5C). In addition, the running endurance of mice has been positively correlated with the abundance of AMP in gut microbiota (Fig. 5D). The related metabolites were enriched in different pathways, including PI3K-Akt signaling pathway, mTOR signaling pathway, FoXO signaling pathway, glycerophospholipid metabolism etc. On the basis of their p value, KEGG annotation was performed (Fig. S4B). Therefore, untargeted metabolomics support the global changes in the composition of the microbiome.

### Graphene-FIR hyperthermia increased phosphorylation of AMPK via GPR 43

**Figure 6:**
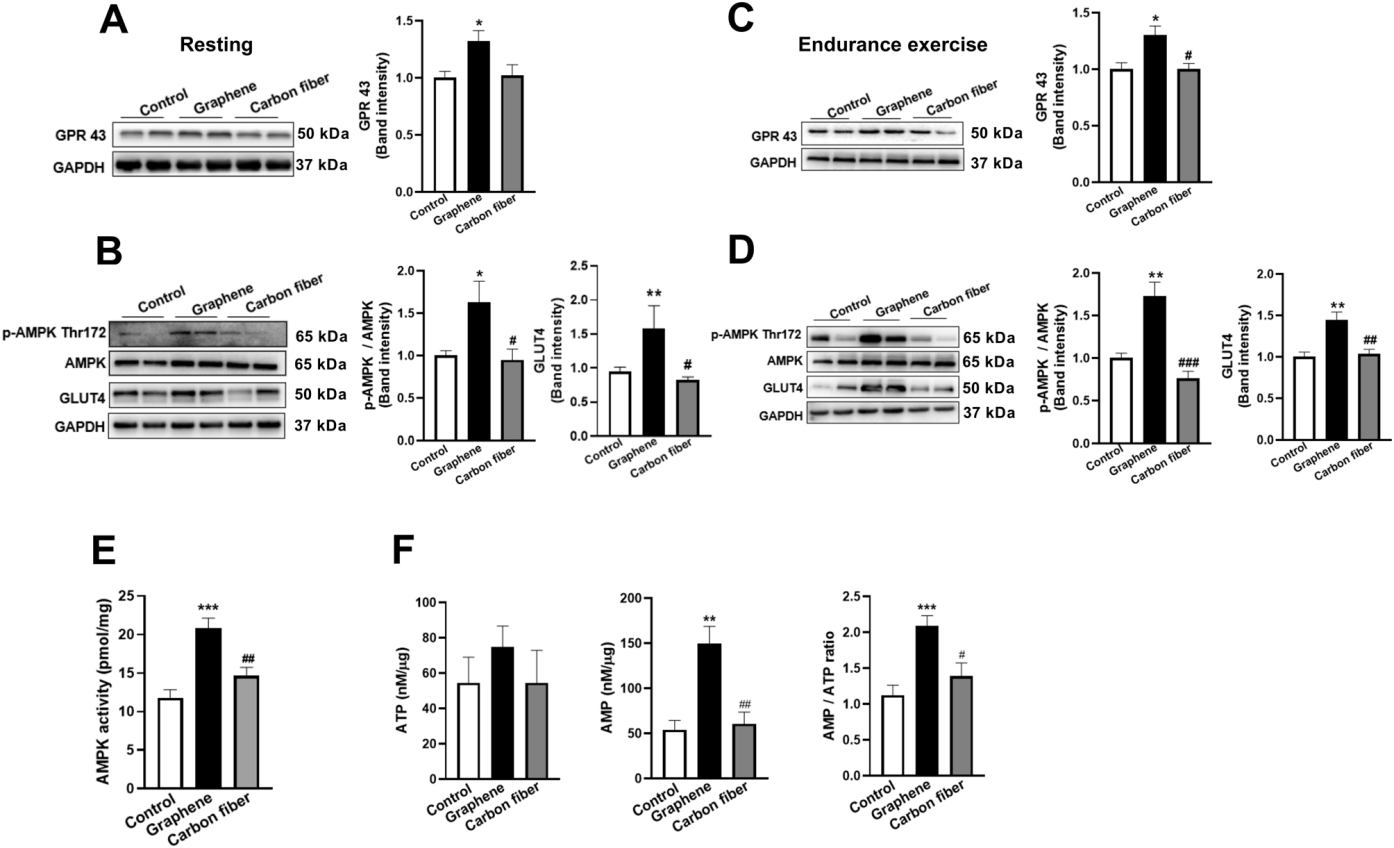
Graphene-FIR hyperthermia increased phosphorylation of AMPK by activated GPR43 in muscle. Exercised and sedentary mice were sacrificed; the gastrocnemius was dissected for Western blot. (**A**) Representative Western blot and quantitation of GPR 43 level in sedentary mice. (**B**) Representative Western blot and quantitation of p-AMPK-Thr172 and GLUT4 level in sedentary mice. (**C**) Representative Western blot and quantitation of GPR 43 level in exercise mice. (**D**) Representative Western blot and quantitation of p-AMPK-Thr172 and GLUT4 level in exercise mice. (**E**) AMPK activity. (**F**) The levels of ATP, AMP and AMP/ATP. Data are expressed as mean ± SEM, n = 3, *p < 0.05, **p < 0.01 and ***p < 0.001 compared to Control group, ^#^*p* < 0.05 and ^##^*p* < 0.01 compared to Carbon fiber group, one-way ANOVA followed by Bonferroni test.

To elucidate the potential roles of microbial metabolism in skeletal muscle function and exercise capacity, we firstly determined the protein expression of GPR43 and p-AMPK Thr172. Compared with control group, GPR43 protein expression increased significantly both in resting and exercise state mice after graphene-FIR treatment, whereas it was not obviously in carbon fiber group (Fig. 6A,C). Previous studies have demonstrated the ability of SCFAs to induce the phosphorylation of AMPK, which is known to be the main regulator of exercise-mediated responses in the skeletal muscle^30^. Therefore, we observed that the expression of p-AMPK (Thr 172) in the graphene group significantly increased compared with the control group, while there was no difference between carbon fiber group and control group (Fig. 6B,D). The AMPK activity of graphene group was significantly higher than the carbon fiber group (Fig. 6E). During exercise the most important glucose transporter is GLUT4, which is rate limiting for glucose uptake into muscle. We found that the expression level of GLUT4 in graphene group was higher than that in controls (Fig. 6B,D). We also measured AMP and ATP levels of muscle in exercise state. The AMP level and AMP/ATP ratio increased in graphene group compared with the respective controls (Fig. 6F). Moreover, the level of AMP and AMP/ATP ratio were decreased in carbon fiber groups compared with the graphene groups. These results indicate that AMPK activated by graphene-FIR might be a key mechanism through which SCFAs exert changes in skeletal muscle metabolism.

### Graphene-FIR hyperthermia mimics exercise by enhanced muscle glucose uptake and blood flow via AMPK

**Figure 7:**
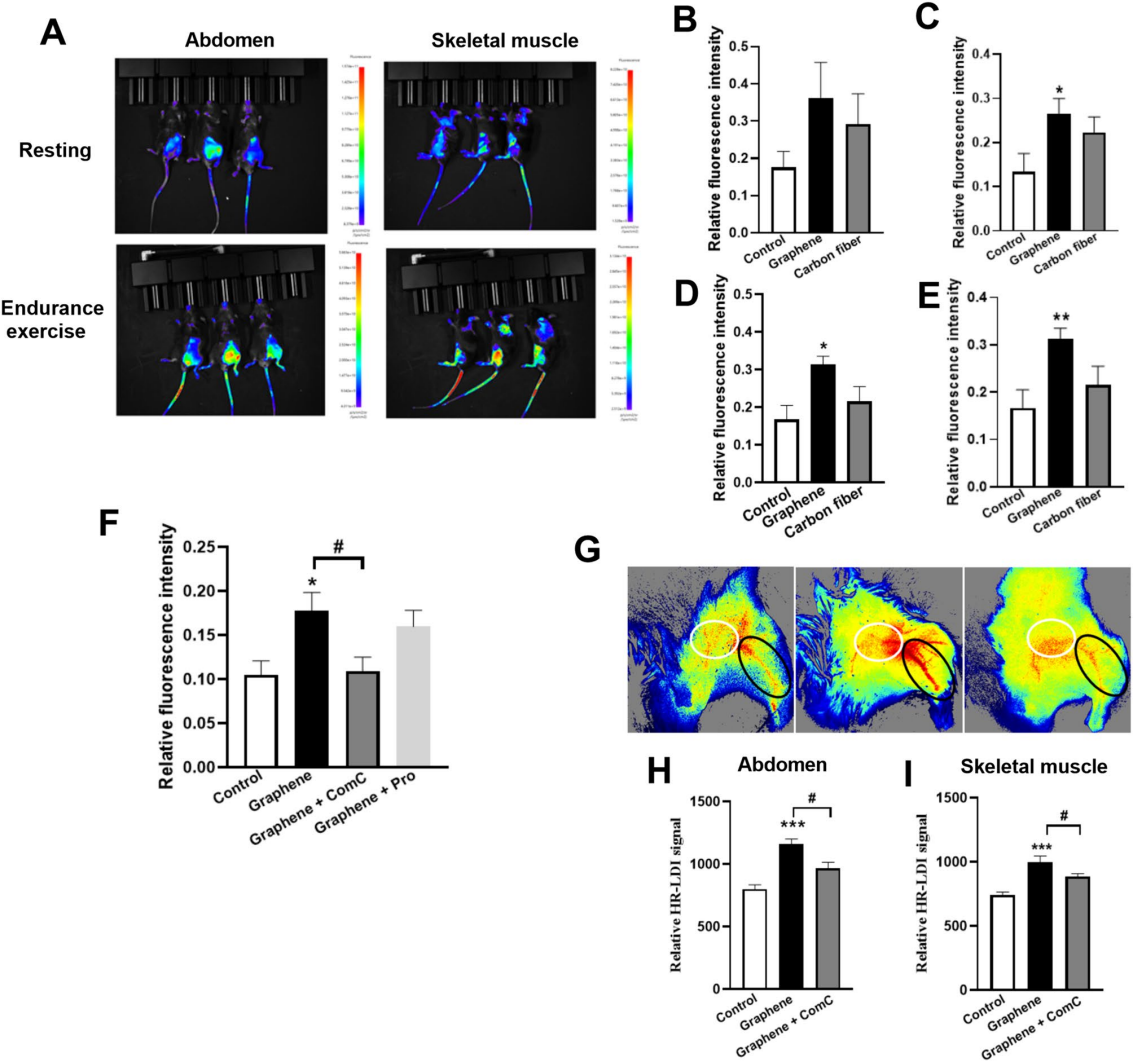
Graphene-FIR hyperthermia mimics exercise by enhanced muscle glucose uptake and blood flow via AMPK. (**A**) Representative fluorescence images showing the glucose uptake rate of mice in abdomen and skeletal. (**B**) Quantitation of relative fluorescence intensity of resting mice in abdomen. (**C**) Quantitation of relative fluorescence intensity of resting mice in muscle. (**D**) Quantitation of relative fluorescence intensity of endurance exercise mice in abdomen. (**E**) Quantitation of relative fluorescence intensity of endurance exercise mice in muscle. (**F**) Quantitation of relative fluorescence intensity of endurance exercise mice in muscle. (**G**) The representative blood flow images. (**H**, **I**) The quantitation of blood perfusion of endurance exercise mice in skeletal abdomen and muscle. Data are expressed as mean ± SEM, n = 5–8, *p < 0.05, **p < 0.05 and ***p < 0.001 compared to Control group, ^#^*p* < 0.05 compared to Graphene group, one-way ANOVA followed by Bonferroni test.

To further verify the effects of graphene-FIR on glucose metabolism, fluorescence imaging was applied to assess the uptake of glucose located in abdomen and skeletal muscle. In the resting state, the glucose uptake ratio of skeletal muscle in graphene group was significantly increased compared to the control group (Fig. 7A,C), and graphene group showed an increasing tendency of glucose uptake in abdomen region (Fig. 7B). However, in the exercise state, graphene-FIR could significantly increase the accumulation of glucose in both muscle and abdomen compared with the control group (Fig. 7D–E). Notably, we found that graphene-FIR induced increased of glucose uptake was blocked by ComC (10 mg/kg, i.p.), but not by propranolol (nonselective β-adrenergic antagonist, 10 mg/kg, i.p.) treatment (Fig. 7F). Interestingly, we also found that graphene-FIR induced increased of blood flow was inhibited by ComC (a specific inhibitor of AMPK, 10 mg/kg, i.p.) systematically administration (Fig. 7G–I).

### Graphene-FIR hyperthermia changed the metabolomic profile of the skeletal muscle

**Figure 8:**
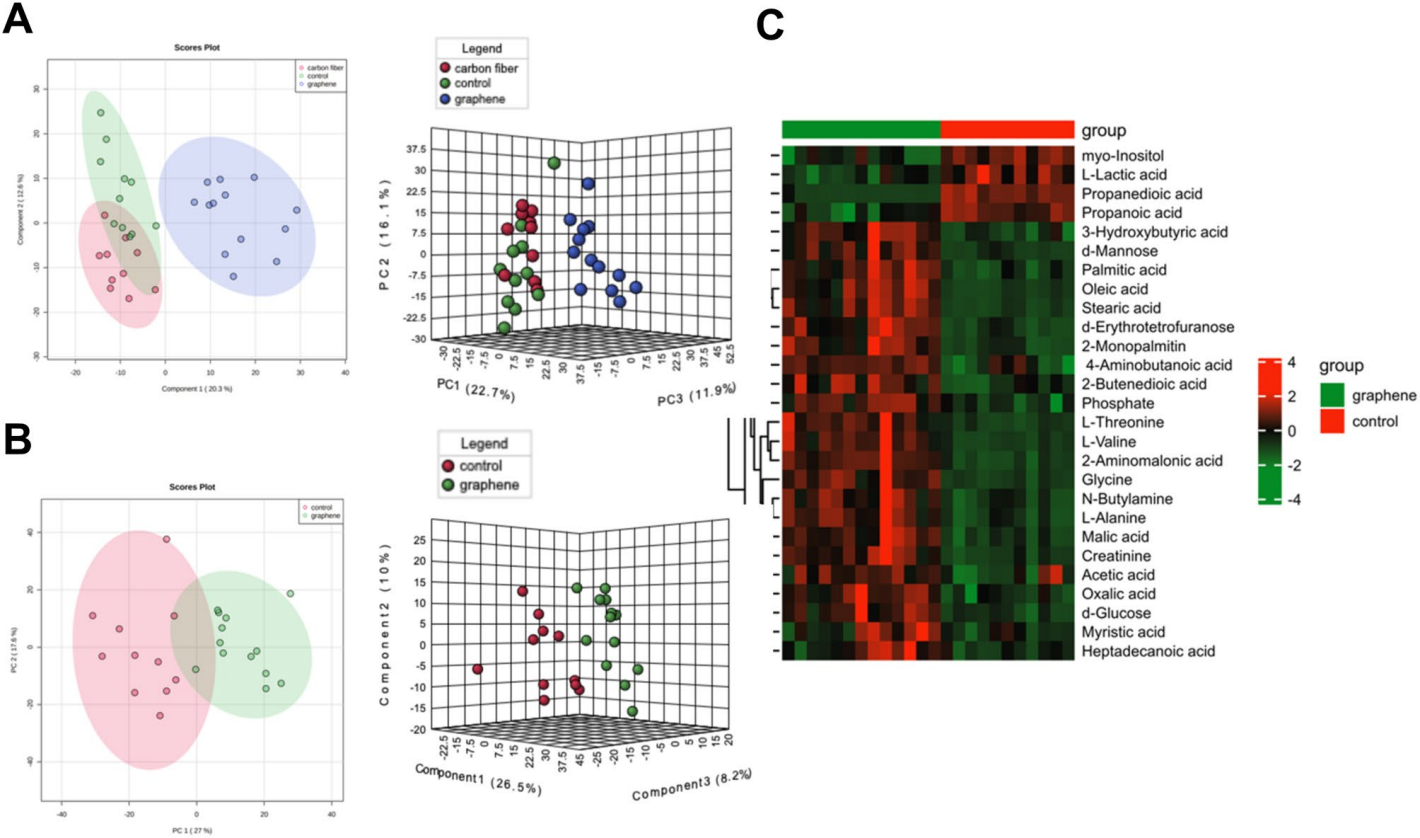
Graphene-FIR hyperthermia changed the metabolomic profile of the skeletal muscle. (**A**) PCA plots of OTUs for different groups. (**B**) PCA plots between the graphene and control groups. (**C**) A heatmap of metabolites in the gastrocnemius of control and graphene groups, red and green represent higher and reduced concentrations of metabolites.

To explore the potential regulatory roles of graphene-FIR on skeletal muscle metabolism, we consider the metabolomic analyses for providing a quantitative profile of metabolites in biological systems. Skeletal muscle samples from exercise mice were analyzed by non-targeted metabolomics. Principal component analysis (PCA) revealed a distinct separation of the graphene group and control group (PC1 22.7%), as shown in Fig. 8A. The Fig. 8B also exhibited obvious separation between the control group and graphene group, indicating that the metabolic profiles of the two groups were completely different. The upregulated metabolites were shown in red in Fig. 8C, whereas the downregulated metabolites were shown in green. Notably, the graphene group's metabolites were significantly altered, with 23 increased (e.g. creatinine and phosphate) and 4 decreased (e.g. lactic acid).

We next investigated the impact of the metabolic pathways involved. As shown in Fig. S5A, multiple pathways including galactose metabolism, glycolysis, gluconeogenesis, fatty acid biosynthesis, butanoate metabolism, and pyruvate metabolism, were shown to be involved in the pathway analysis of the 25 ANOVA-significant metabolites. Pathway enrichment analysis identified a nearly fivefold enrichment in galactose metabolism, fatty acid biosynthesis, pyruvate metabolism, glycolysis/gluconeogenesis, butanoate metabolism, propanoate metabolism, and alanine metabolism pathways with p values ~ 1 × 10^−3^ (Fig. S5B).

### Graphene-FIR hyperthermia rescued the running endurance of ABX mice

**Figure 9:**
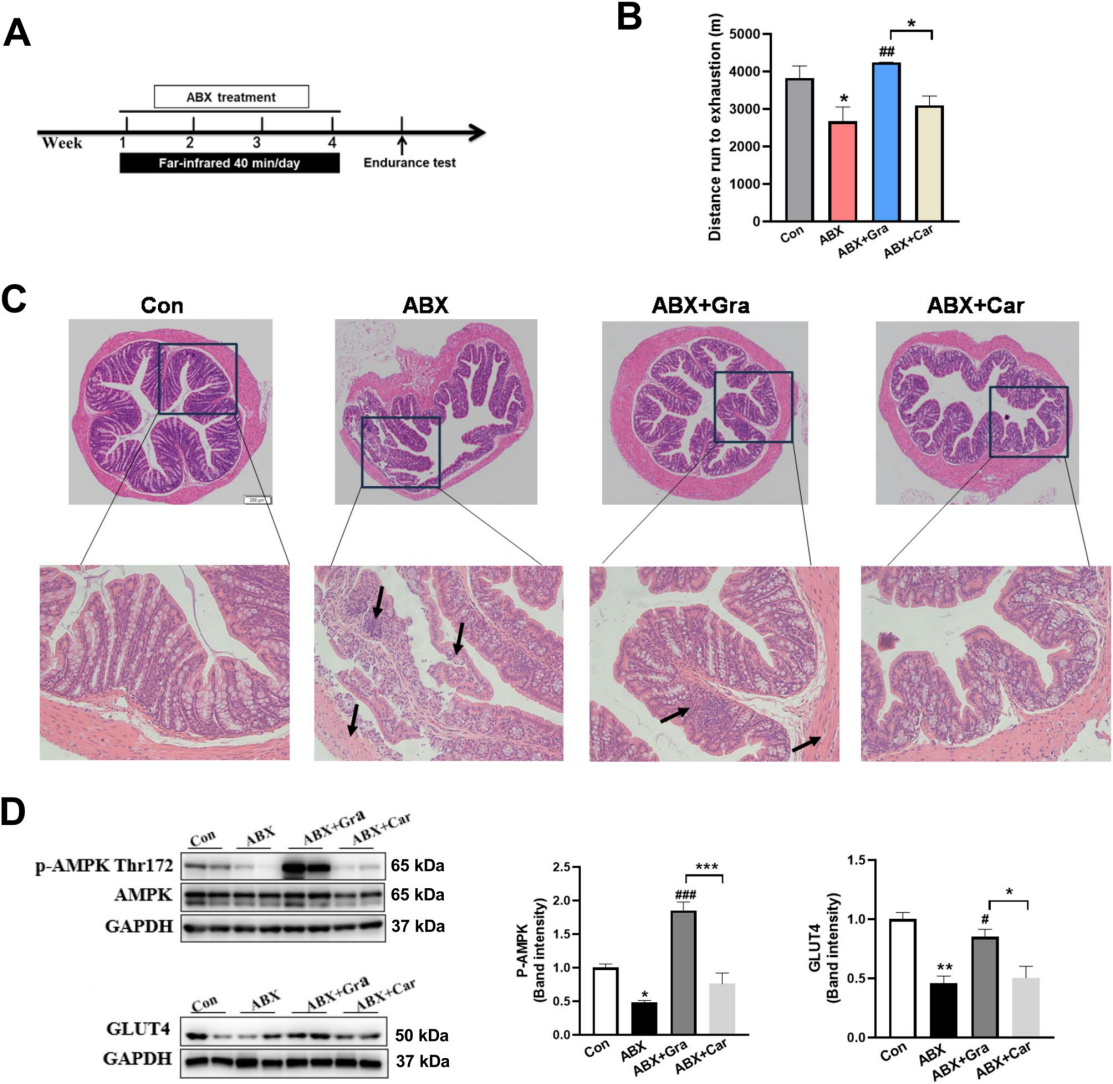
Graphene-FIR hyperthermia rescued the running endurance of ABX mice. (**A**) Experimental procedure. (**B**) Running distance until exhaustion on the treadmill for control, ABX, ABX + Gra and ABX + Car groups (n = 8). (**C**) H&E staining images of colon tissues were presented. The morphology changes of the intestinal mucosa, crypts of the intestinal mucosa and muscular layer were indicated by arrows. (n = 3–5, scale bar = 200 μm, × 40). (**D**) Representative Western blot and quantitation of p-AMPK-Thr172 and GLUT4 level in gastrocnemius (n = 3). Data are expressed as mean ± SEM, *p < 0.05, **p < 0.01 compared to Con group or ABX + Car group, ^#^*p* < 0.05, ^##^*p* < 0.01 and ^###^*p* < 0.001 compared to ABX group, one-way ANOVA followed by Bonferroni test.

To further demonstrate the role of the microbiome in endurance-exercise promoted by graphene-FIR hyperthermia, we administered an antibiotic cocktail (ABX) in the drinking water for 4 weeks (Fig. 9A). The body weight and food intake were not changed in different groups (Fig. S6A,B, Supporting Information). Microbiota depletion significantly changed the morphology of the small intestine. The small intestine of ABX mice was significantly longer than control mice (Fig. S6C, Supporting Information). The morphology of the fecal pellets was also significantly changed (Fig. S6D, Supporting Information). Graphene-FIR significantly decreased the length of small intestine, and reduced the weight of fecal pellets compared to the ABX mice. Histological analysis showed that the intestinal mucosa of the mice in the ABX group was atrophied, and the crypts of the intestinal mucosa were shallower, muscular layer were thinner. The damaged morphology of intestinal were partly improved by graphene-FIR hyperthermia (Fig. 9C).

Moreover, treadmill running time was lower in the ABX group than the control group, graphene-FIR hyperthermia significantly increased treadmill running time, while the carbon-fiber group only have an increasing trend (Fig. 9B). As shown in Fig. 9D, the protein level of p-AMPK was significantly lower in ABX mice in skeletal muscle, which is consistent with their impaired ability to maintain endurance. Graphene-FIR was significantly up-regulated p-AMPK and GLUT4 expression compared to the ABX group, and significant difference was also observed between carbon fiber and graphene-FIR mice. These data indicated that the effect of graphene-FIR hyperthermia on exercise endurance may attribute to rescue the microbiota depletion and AMPK activity.

## Discussion

With the advancing innovation of technology, specialty pure FIR emitting, have become safe, effective, and widely used in clinical treatment. However, FIR application in physical activity and metabolism requires understanding the potential mechanisms of biological effects due to FIR radiation at specific range. In our study, we introduced a graphene-based flexible device tailored for systemic FIR hyperthermia therapy. The graphene-based device showed higher electrothermal stability and resonance effect in hyperthermia therapy compared to the carbon fiber-based device. We hypothesized that graphene-FIR hyperthermia could promote exercise capacity as exercise mimetic. Our research provided a mechanistic insight into the biological effects of graphene-FIR radiation within a specific wavelength range. The resonance effects generated by graphene-FIR improved the body temperature and glucose uptake which associated with the metabolic benefits. We suggested that the application of graphene-FIR stimulates the metabolism homeostasis-related AMPK/GLUT4 signaling pathway through gut-muscle interaction. The findings of the present study provided evidence that improving physical activity and metabolisms with graphene-FIR hyperthermia as exercise mimic, and the possible future applications in medical are wide ranging.

In recent years, graphene and its unique properties have been widely used in biomedical field. In the present study, owing to its high carrier mobility, conductivity, flexibility and optical transparency, a manufactured low-voltage, flexible graphene film was utilized as a FIR emitter. We demonstrated that exposure to graphene-FIR promoting running endurance in animal experiments. Graphene-FIR hyperthermia significantly improved the running distance of mice both in the low and high exercise intensity. We excluded for the possible psycho-stimulatory effect after long-term graphene-FIR hyperthermia, in according to the locomotor activity test.

However, the question that arises is: how the graphene-based photo-biomodulations work in the enhancement of running endurance? To investigate the spectral properties and metabolic regulatory effects of graphene-based FIR, a carbon fiber-based device was employed as a control. We found that the emission peak wavelength of the graphene-based device aligned more closely with the human body's absorption peak compared to the carbon-fiber device. Graphene-FIR hyperthermia was effective in raising both core and epidermal temperatures in mice. Remarkably, these thermal effects persisted for almost 1 h after the graphene-FIR heater was removed. Resulting epidermal temperature was higher when the skin was irradiated with graphene-FIR than if similar thermal loads from shorter wavelengths were used. It indicated that graphene-FIR hyperthermia promoted metabolism processing. Graphene-FIR hyperthermia increased blood flow in femoral muscle and abdomen region. The microvascular blood flow plays an important role in muscle metabolism and exercise capacity. Blood flow to the working muscles is crucial for muscle function during exercise^31^. Skeletal muscle microvascular blood flow is stimulated by muscle contraction to aid in the transport of nutrients and hormones^32^. The efficient transport of radiant energy to the tissues may have been made possible by the graphene-FIR's ability to more effectively resonance with cellular frequencies. Notably, exercise enhances endurance capacity in humans, improves heart function, and stimulates peripheral blood flow. Additionally, AMPK activation in muscles increases muscle vasodilation and microvascular perfusion^33,34^. Our results demonstrated that graphene-FIR can improve blood flow in femoral muscle and abdomen, and these effects could be inhibited by AMPK inhibitor. These provided an important foundation for the biological effects by graphene-based FIR hyperthermia except for its thermo-effect. However, whether graphene-FIR hyperthermia can play a similar role as exercise-mimetics to improve exercise capacity and metabolisms by AMPK activation, and the fundamental mechanism is still unknown.

The composition and diversity of gut microbiota have impact on intestinal function and health status^35^. Previous studies have demonstrated that endurance training can influence the composition and diversity of the gut microbiota in mice^36^. Human microbiome studies have also shown that the gut microbiota of athletes is more diverse as compared with that of controls^37^. In the present study, we found that graphene-FIR hyperthermia significantly increased the α- and β-diversities index of gut microbiota compared with the control group. The normal gut microbiota comprises of two major phyla, Bacteroidetes and Firmicutes, comprise 90% of the average gut microbiota^38^. A recent study showed that compared to non-athletes, athletes exhibited higher levels of firmicutes and lower levels of bacteroidetes^39^. The microbiota of exercise mice presented a greater abundance of firmicutes and lower bacteroides, with related changes highly correlated with VO_2_ max^37,40^. We found that the Bacteroidetes/Firmicutes ratio was significantly decreased in graphene-FIR group compared with the controls. Furthermore, our findings demonstrated a significant increase in several potentially beneficial bacterial genera in mice exposed to graphene-FIR radiation, specifically *Blautia*, *Allobaculum*, and *Anaerostipes*^41–43^. *Allobaculum* and *Blautia* are known producers of short-chain fatty acids (SCFAs), which are conducive to a healthier gut environment^41^. It has been reported that *Allobaculum* were enriched in the exercise animals^41,42^. Dietary inositol could converse into propionate and acetate by *Anaerostipes*, which have a potential beneficial role for promoting host health^43^. Our results also indicated that the running endurance of mice was significantly positively correlated with the abundance of *Anaerostipes* and *Blautia*. Graphene-FIR hyperthermia led to significant changes of fecal metabolic phenotype. The abundance of AMP was increased in graphene group, which was strongly correlated with running endurance. These results indicated that graphene-FIR hyperthermia can positively alter the amount and structure of the gut and enriched the SCFAs-producing bacteria, which was associated with improving the exercise endurance.

Most current studies generally believe that moderate exercise training is beneficial to gut microbiota^37^. In contrast to light exercise, strenuous exercise cause changes in immune response by reducing the gastrointestinal blood flow while increasing permeability of the gastrointestinal epithelial wall and the destruction of gut mucous thickness^44^. In our study, we found that graphene-FIR increased the blood flow and the abundance of intestinal probiotics, exerted an intestinal protective effect. These protective effects of gut microbiota may help delay the fatigue symptoms in endurance performances. Therefore, the graphene-FIR group showed greater exercise endurance in acute strenuous exercise tests.

Numerous studies have demonstrated that gut microbiota impact the function of various organs through bacterial metabolites, such as SCFAs and bile acids^45^. Growing evidence suggests that there is bidirectional contact between the gut microbiota and muscle, and some researchers have proposed the existence of a "gut-muscle axis"^23,46,47^. It has been documented that SCFAs may stimulate AMPK to phosphorylate in skeletal muscle^28^. It is significant to note that AMPK activation also plays a role in energy homeostasis through the processes of fatty acid oxidation, glucose absorption, glycogenesis, and glycolysis^48^. Studies conducted in vitro have shown that butyrate treatment improves fatty acid absorption in muscle cells^49^. The glucose transporter protein, GLUT4, is essential for the uptake and metabolism of glucose in skeletal muscle^50^. It is suggested that the influence of SCFAs on GLUT4 expression may promote glycogen restoration and glucose uptake^51^. Notably, the administration of acetate has been shown to accelerate the rate of skeletal muscle glycogen replenishment in rats following exercise^52^. In present study, graphene-FIR stimulated the phosphorylation of AMPK and GLUT4 expression, and increased the AMP/ATP ratio in gastrocnemius muscle, probably because of the ability of GPR43 activation to increase AMP concentrations within skeletal muscle tissue. We also found that graphene-FIR induced increased of glucose uptake was blocked by the AMPK inhibitor, but not by the β-adrenergic antagonist. It was suggested that graphene-FIR promotes glucose uptake by activating AMPK.

AMPK is activated by numbers of activators, including the AMP mimicking AICAR. Previous study demonstrated that AICAR can induce metabolic changes and improve running endurance without exercise training^27^. In our study, we speculated that graphene-FIR could restore a more favourable energy balance in gut-muscle axis and improved the running endurance through AMPK activation. Metabolomic analyses metabolites were significantly altered after graphene-FIR hyperthermia. We found the abundance of AMP in gut microbiota was increased in the graphene group and it has positively correlated with running endurance. In muscle metabolomics analysis, graphene-FIR hyperthermia decreased the lactic acid and increased the creatinine and phosphate. Metabolic pathways analyses revealed that galactose metabolism, glycolysis, gluconeogenesis, fatty acid biosynthesis, butanoate metabolism and pyruvate metabolism were involved in graphene-FIR hyperthermia. Therefore, AMPK phosphorylation may be a key mechanism by which graphene-FIR exert changes in gut-muscle axis metabolism.

To further verified the impact of graphene-FIR on exercise capacity and glucose metabolism through the modulation of microbiota homeostasis and AMPK activation, we utilized ABX mice as an animal model for gut microbial dysbiosis. It has been reported that gut microbiota depletion via antibiotics impaired endurance exercise performance and skeletal muscle contractile function in mice. Natural reseeding of the mice improved skeletal muscle function and restored the gut microbiota^20,53,54^. Our results indicated that the decreased of running endurance in ABX mice was reversed by graphene-FIR hyperthermia. We also observed that the expression level of p-AMPK and GLUT4 was up-regulated in graphene-FIR group compared to ABX group. Therefore, the effect of the graphene-FIR on skeletal muscle metabolism and especially on the endurance capacity may attribute to regulation of energy homeostasis in the gut microbiota.

## Conclusions

Collectively, our findings demonstrated that the graphene-based flexible device was different from carbon fiber materials, with single-layer structure and unique electrothermal stability and spectral properties. Graphene-FIR hyperthermia could promote exercise capacity and glucose metabolism via gut-muscle interaction. The potential mechanism may involve the modulation of microbiota homeostasis and activating AMPK. We present evidence supporting a novel application that graphene-based FIR hyperthermia can be used to as exercise mimetic in the future.

### Supplementary Information

Supplementary Information.

## Data Availability

All data used/analyzed during the current study are available from the corresponding author on reasonable request.
