# Supplementary material for: Far-infrared therapy promotes exercise capacity and glucose metabolism in mice by modulating microbiota homeostasis and activating AMPK
Source: Sci Rep. 2024 Jul 15;14:16314. doi: 10.1038/s41598-024-67220-5 (PMC11251280; doi:10.1038/s41598-024-67220-5)

**Supporting Information**

**Far-infrared therapy promotes** **exercise capacity and glucose metabolism in mice by** **modulating microbiota homeostasis and activating AMPK**

**Shuo Li^a^, Xiao-yao Miao^b^, Jin-shui Zhang^c^, Dong-dong Wei^a^, Hua-jin Dong^a^, Rui Xue^a^, Jing-cao Li^a^, Yang Zhang^a^, Xiao-xing Feng^d^, Jin Li^a^, You-zhi Zhang^a*^**

^a^State Key Laboratory of Toxicology and Medical Countermeasures, Beijing Key Laboratory of Neuropsychopharmacology, Beijing Institute of Pharmacology and Toxicology, Beijing, China

^b^Department of Pharmaceutical Science, Beijing Institute of Radiation Medicine, Beijing, China

^c^School of Medicine, Anhui University of Science and Technology, Huainan 232001, China

^d^Grahope New Materials Technologies Inc. Shenzhen 518063, China

Shuo Li and Xiao-yao Miao made equal contribution to the work.

^*^Correspondence: bcczyz@163.com（YZ Zhang）

1. **Supplementary Figures**

**
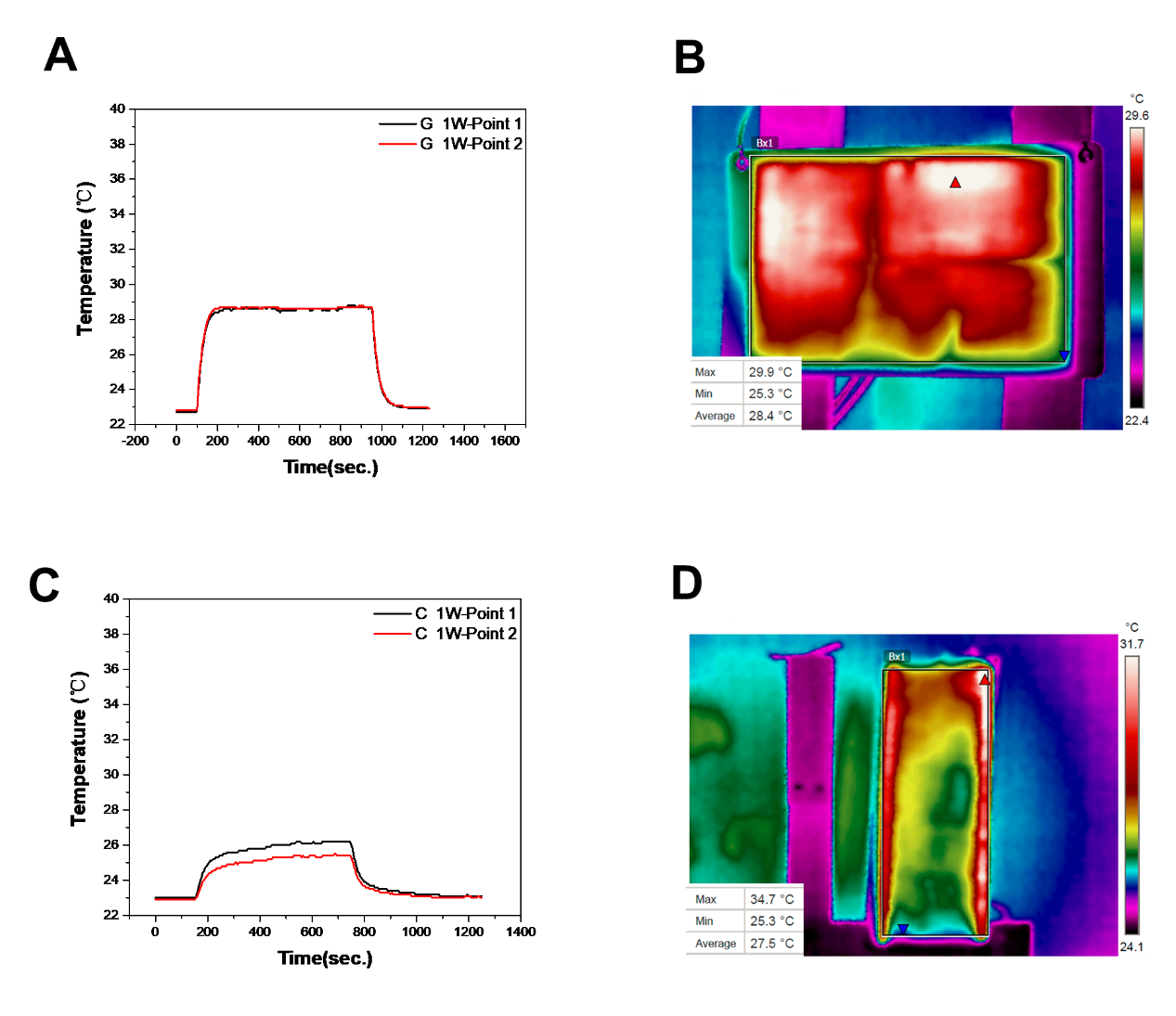
**

**Figure S1. Electrothermal characterization of graphene-based and carbon fiber-based device.** (A, B) Infrared thermal imaging of the graphene-based device and temperature-time curve. (C, D) Infrared thermal imaging of the carbon fiber-based device and temperature-time curve. (Input power: 1 W for the graphene-based device and carbon fiber-based device).


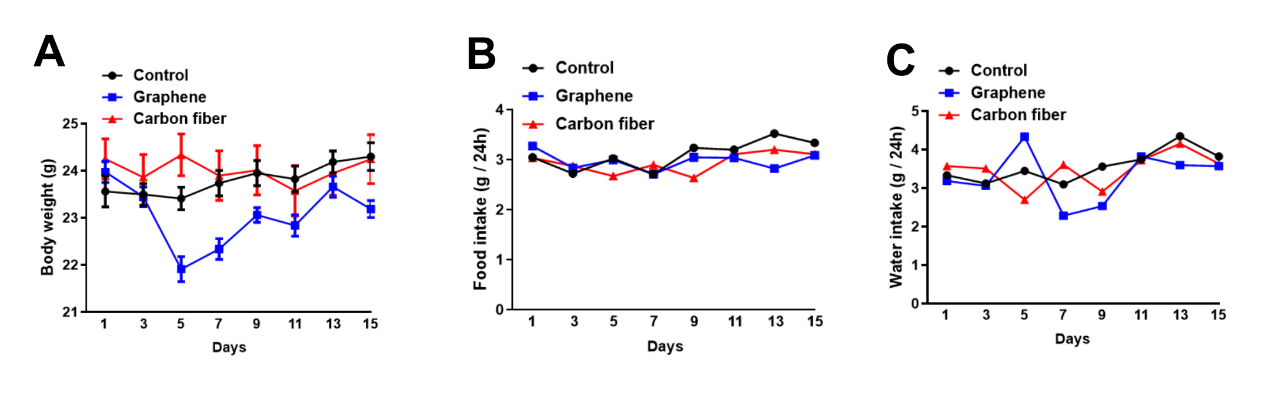


**Figure S2. The** **body weight, food intake and water intake changes of mice throughout FIR hyperthermia period.** (A) body weight. (B) food intake. (C) water intake.


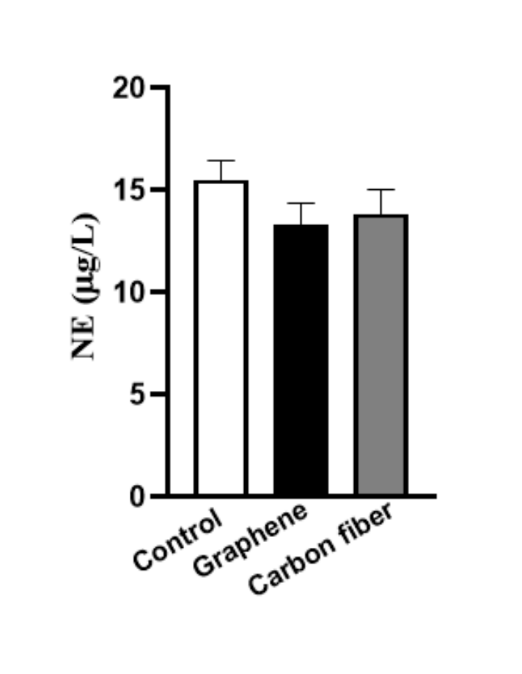


**Figure S3. Effect of FIR hyperthermia on the serum NE level in mice.** Data are expressed as mean ± SEM; n = 8 in each group, one-way ANOVA followed by Bonferroni test.


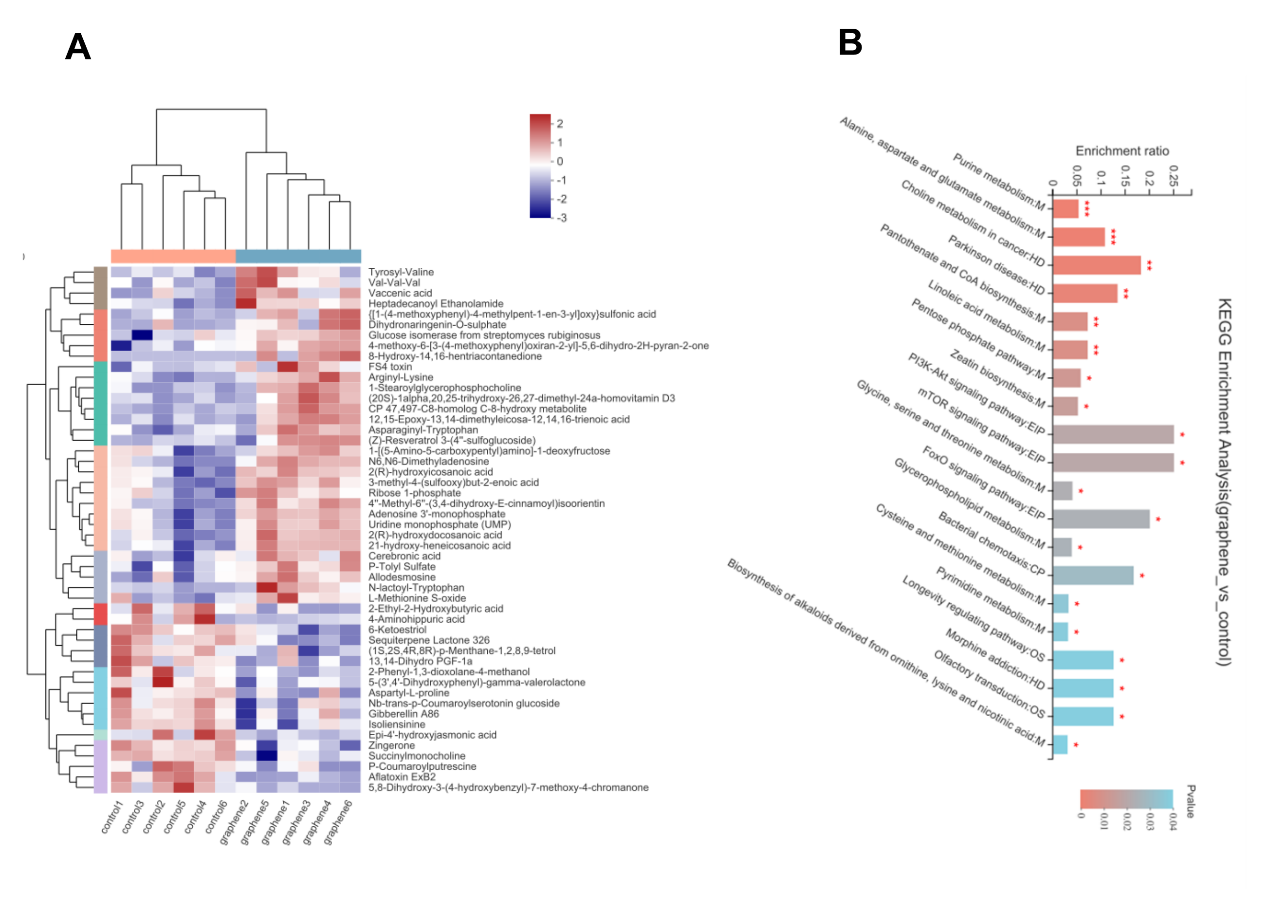


**Figure S4. Graphene-FIR hyperthermia changes the metabolomic profile of the gut microbiome.** (A) Heat map of the significantly changed metabolites identified in UPLC-Q-TOF/MS data of fecal samples. (B) KEGG enrichment analysis in graphene versus control group [54, 55, 56]. n = 6 in each group.


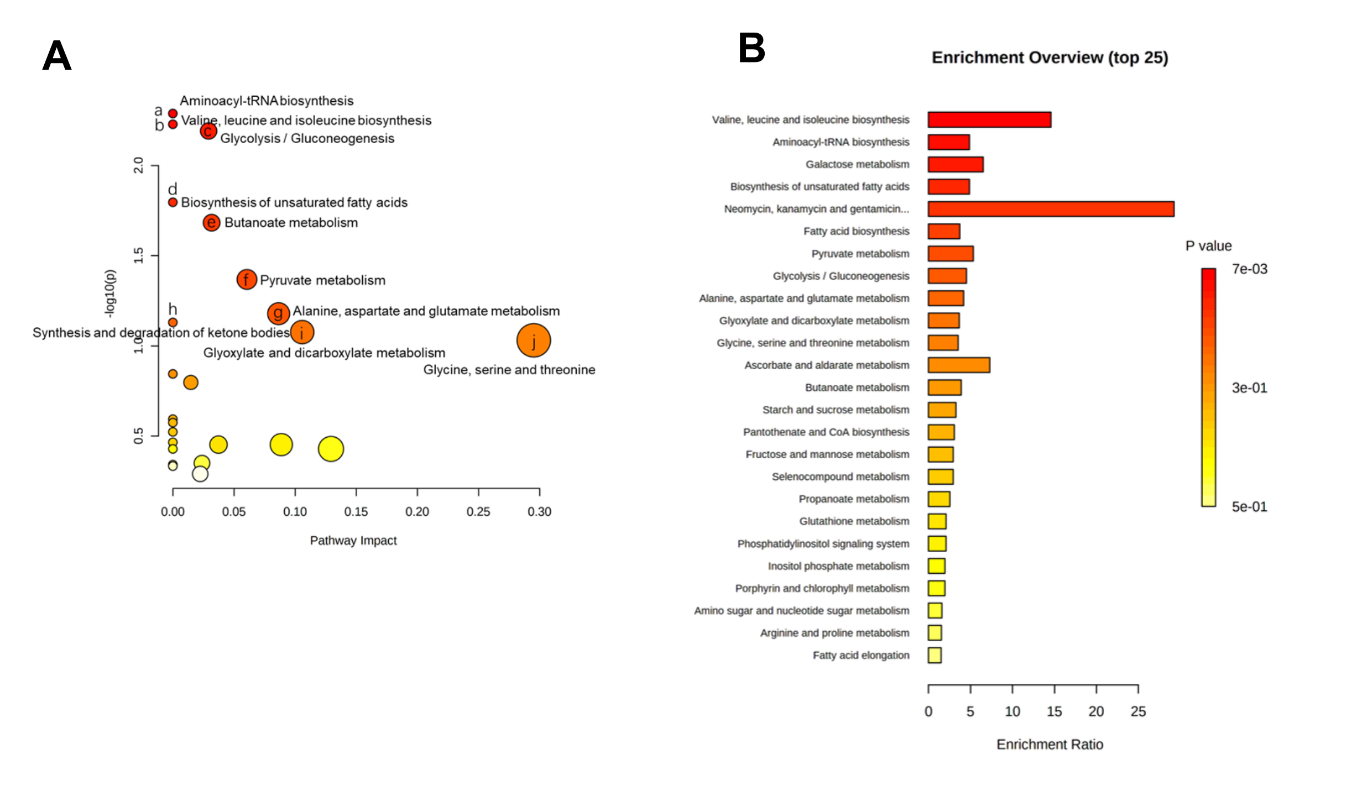


**Figure S5. Graphene-FIR hyperthermia changed the metabolomic profile of the skeletal muscle.** (A) The bubble map of pathway analysis of metabolites. (B) Pathway enrichment map of metabolites [54, 55, 56].


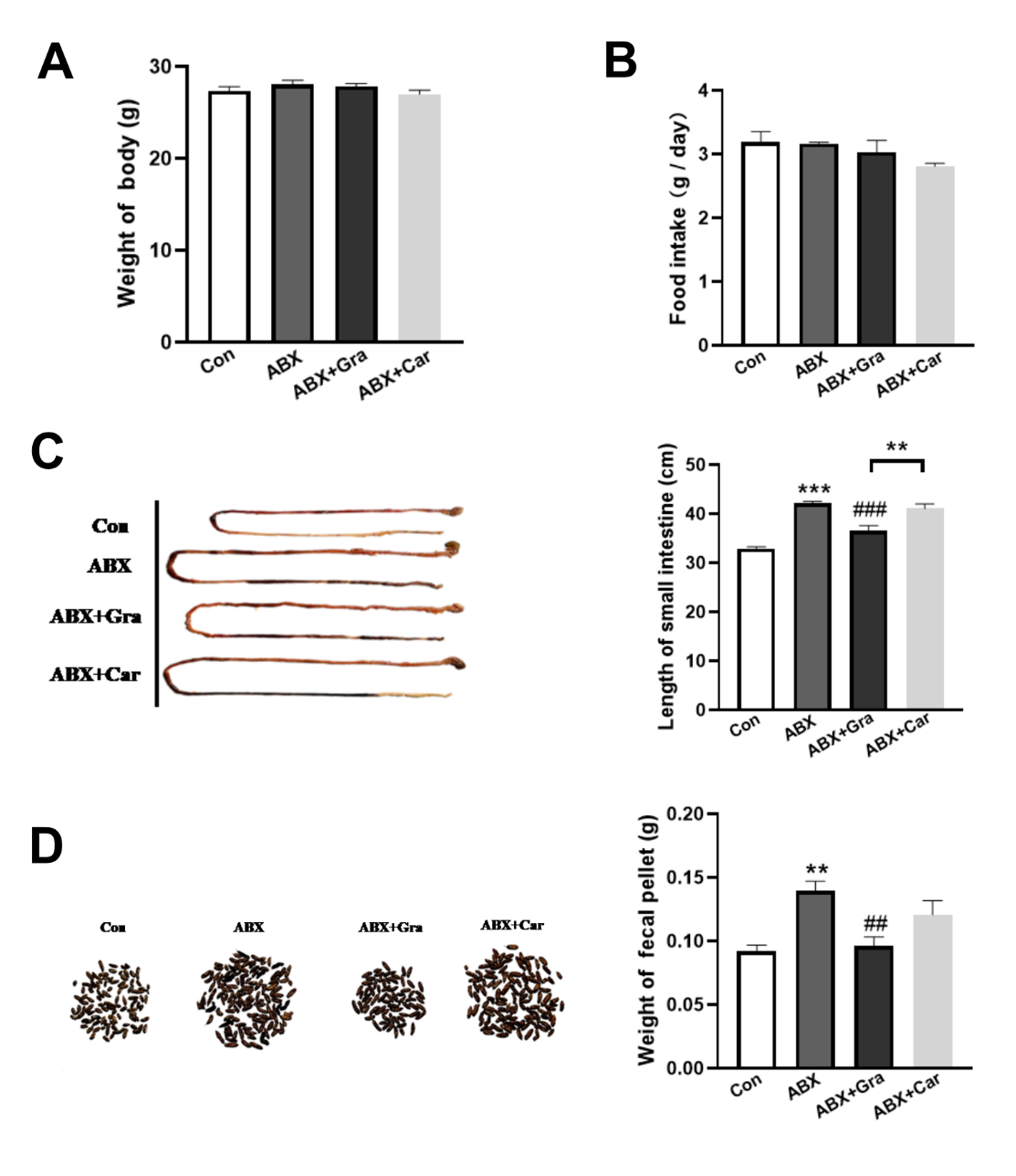


**Figure S6. Effect of graphene-FIR hyperthermia on the small intestine and fecal pellet of ABX mice.** (A) The weight of the mice. (B) The food intake of the mice. (C) The morphology and length of the small intestine. (D) The morphology and weight of the fecal pellet. Data are expressed as mean ± SEM; n = 8 in each group, ^**^*p*<0.01 and ^***^*p*<0.001 compared to Con group, ^##^*p*<0.01 and ^###^*p*<0.001 compared to ABX group, one-way ANOVA followed by Bonferroni test.

| Experiment | Grouping | Timepoints | Number of samples | Conditions and treatments |
| --- | --- | --- | --- | --- |
| Exercise endurance test (Fig. 2D-E) | Control, graphene, carbon fiber | On the day 14 | 8 | Mice received 40 min/day FIR radiation for 14 days. On day 15, mice received the endurance test. |
| Locomotor activity (Fig. 2F) | Control, graphene, carbon fiber | On the day 14 and 15 | 8 | Mice were tested 1 h or 24 h after the last FIR irradiation. |
| Core temperature test (Fig. 3C) | graphene, carbon fiber | On the day 15 | 8 | The core temperature of mice was tested before and after radiation. |
| Epidermal temperature test (Fig. 3D-E) | Graphene, carbon fiber | On the day 15 | 8 | The Epidermal temperature of mice was tested before and after radiation. |
| Blood ﬂow test (Fig. 3F-G, Fig. 3G-I) | Control, graphene, graphene+ComC,  carbon fiber | On the day 15 | 8 | The blood ﬂow was measured without acute FIR irradiation. |
| Glucose uptake test (Fig. 7A-F) | Control, graphene, graphene+ComC,  graphene+Pro | On the day 15 | 5-8 | The glucose uptake was measured without acute FIR irradiation. |
| Exercise endurance test for ABX mice (Fig. 9B) | Control, ABX, ABX+Gra,  ABX+Car | On the day 29 | 8 | Mice received ABX treatment and FIR radiation for 4 weeks before the endurance test |

**Table 1. Grouping information.** The grouping information of different animal experiments.

1. **Supplementary materials and methods**

**2.1. Materials**

HPLC-grade methanol and acetonitrile were purchased from Fisher Scientific. Methoxyamine, pyridine, and n-methyl-n-(trimethylsilyl) trifluoroacetamide (MSTFA) with 1% trimethylchlorosilane (TMCS) were purchased from Sigma. Ultrapure water was purified by a Milli-Q water purification system from Millipore. CY5.5-Glucose dye was purchased from Qiyue Biology (Xian China) dissolved in 5% DMSO. AMPK assay kit was purchased from Genmed Scientifics Inc. (USA). ATP assay kit was purchased from Beyotime (Shanghai, China). AMP assay kit was purchased from Light Ace Lab CO., LTD (Beijing, China). Anti-GPR 43 antibody was purchased from Santa Cruz. The following antibodies were purchased from Cell Signaling Technology: anti-p-AMPK Thr172 (1:1000), total AMPK (1:1000), GLUT4 (1:1000), GAPDH (1:5000). All secondary antibodies conjugated with horseradish peroxidase (HRP) were purchased from Santa Cruz Biotechnology. Enhanced chemiluminescent solution (ECL) were obtained from Pierce.

**2.2. Infrared spectrum characterization and analysis**

The infrared spectrum of FIR fiber was detected by using a Fourier transform infrared spectrometer (Thermo Fisher, Nicolet iS50). The room temperature was maintained at 25 °C during the procedure. The ﬁnal spectrum was recorded by wavelength. The optical probe assembly was connected to the Fourier infrared spectrometer equipment via a coupler. Then, put the probe in the air to collect background data first and then kept close to the surface of the FIR fiber for collect data. The obtained data were processed by advanced ATR correction and the calculation formula is

out = (in − min (in)) ∕ (max (in) − min (in))

Pearson's correlation coefficients (PCCs) and Euclidean distances were used to assess the similarity between different spectra. The PCC is deﬁned as follows:

$$\rho_{x,y}=\frac{cov(x,\mathsf{y})}{\sigma_{x}\sigma_{\mathsf{y}}}=\frac{E[\left( x-\mu_{x} \right)\left( \mathsf{y-}\mathsf{y}_{x} \right)]}{\sigma_{x}\sigma_{\mathsf{y}}}$$

Euclidean distance is a commonly used distance deﬁnition that refers to the true distance between two points in m-dimensional space. The Euclidean distance is deﬁned as follows:

$$d\left( A,B \right)\boldsymbol{=}\sqrt{{\boldsymbol{(}a_{1}{-b}_{1}\boldsymbol{)}}^{\boldsymbol{2}}\boldsymbol{+}{\boldsymbol{(}a_{2}{-b}_{2}\boldsymbol{)}}^{\boldsymbol{2}}\boldsymbol{+\ldots+}{\boldsymbol{(}a_{n}{-b}_{n}\boldsymbol{)}}^{\boldsymbol{2}}}$$

The smaller the Euclidean distance between the vectors, the greater the similarity. The similarity between vectors A and B is deﬁned as follows:

$$E_{A,B}=\frac{1}{1+d(A, B)}$$

**2.3. Exercise endurance test**

Prior to exhaustion running, mice were pre-adapted to the treadmill for 10 min per day for 3 days. Mice unable to complete training were excluded. On the test day, set the electric shock intensity appropriately 2.55 mA and treadmill protocol: 14m/mim(2min), 16m/mim(3min), 18m/mim(25min), 20m/mim(15min), 22m/mim(15min), 24m/mim(15min), 26m/mim(15min), 28m/mim(90min). Intervene with a wire brush tap or tail tickle if mice remain in the fatigue zone. Remove mice staying for over 5 seconds and log the run time and distance.

**2.4. Temperature-time curve testing**

The temperature-time curve of graphene and carbon fiber-based device were detected by the TOPRIE TP700 Multiplex Data Logger (Shenzhen, China). Briefly, tests were conducted in a controlled 25°C room without windows. Temperatures of the heating films at two distinct points were monitored under constant power, with real-time data captured and plotted with time on the X-axis and temperature on the Y-axis.

**2.5.** **16S rRNA gene sequence analysis**

The fecal samples were collected from the colon during sacrifice. The collected samples were immediately stored at −80 °C for DNA extraction. Changes in gut bacterial composition were measured by sequencing of the bacterial 16S rRNA gene. The bacterial DNA was extracted using the PowerMax Soil DNA Isolation Kit (MO BIOLaboratories, Carlsbad, CA, USA) following the manufacturer’s instructions. DNA concentration and purity were monitored on 1% agarose gels. The extracted DNA was stored at −80 °C prior to 16S rRNA sequencing. The V3-V4 hypervariable region of the 16S rRNA gene was ampliﬁed by PCR using the 341F (5′-CCTACGGGNGGCWGCAG-3′) and 805R (5-GACTACHVGGGTATCTAATCC-3′) barcoded primers. A pair-end library (insert size of 450–470 bp for each sample) was constructed using the TruSeq DNA PCR-Free Sample Preparation Kit (Illumina, San Diego, CA, USA), and high-throughput sequencing was performed on an Illumina HiSeq2500 platform. The raw 16S rRNA gene sequencing reads were demultiplexed, quality-filtered by fastp version 0.20.0 and merged by FLASH version 1.2.7. Operational taxonomic units (OTUs) were clustered at a 97% similarity cutoff using UPARSE version 7.1, and any chimeric sequences were identified and removed. The taxonomic classification of each representative sequence from the OTUs was performed using RDP Classifier version 2.2, against the 16S rRNA database, with a confidence threshold of 0.7.

**2.6. GC/MS for skeletal muscle metabolic profiling**

Skeletal muscle samples were flash frozen in liquid nitrogen, weighed (30-50 mg wet wt), then placed in buffer (dichloromethane:methanol:water, 2:5:2) at a standard concentration of 30 mg/400 μL buffer and fully homogenized on ice for 20-25 s. The tissue homogenate was centrifuged at 12000 rpm for 15 min at 4 °C. 200 µL supernatant was transferred to the GC vial. Then 500 µL of methanol was added to the deposit and the mixture was vortexed for 2 min. Afterward, the mixture was ultrasonically extracted for 15 min, followed by centrifugation (12,000 rpm) for another 15 min. Then, another 200 μL supernatant was transferred to the GC vial and evaporated to dryness under a stream of nitrogen gas. Metabolites were made volatile with 50 µL MSTFA with 1 % TMCS at 70 °C for 1 h, After 100 µL of n-heptane was added and mixed, the mixture was centrifuged at 3000 rpm for 10 min. Then, the supernatant was transferred to the GC microvial for analysis. QC sample was obtained by mixing aliquots of all samples and then analyzed using the same method with the analytic samples. And the QC data was used to assess the system stability.

GC/MS analysis was carried out using a SHIMADZU GC-MS-2010 Plus series equipped with an Rxi-5ms capillary column (30 m × 0.25 mm, 0.25 μm). The temperature of the GC oven was performed as follows: the initial temperature was maintained at 80 °C for 5 min, and then raised to 280 °C at a rate of 4 °C per minute. The mass spectrometer was operated in the EI mode (70 eV), and the quadrupole was 150 °C. Data acquisition was performed in a full scan mode with repetitive scanning from 60 m/z to 600 m/z for 1 s. The ion source temperature was 230 °C, interface temperature was 280 °C.

**2.7. LC/MS for** **fecal metabolic profiling**

For metabolite extraction, fecal samples were weighed (50mg) and metabolites were extracted using a 400 µL solution of methanol:water (4:1, v/v). The mixture was allowed to settle at -20℃ and then treated with the High Throughput Tissue Crusher Wonbio-96c (Shanghai Wanbo Biotechnology Co., Ltd) at 50 Hz for 6 minutes. It was followed by vortexing for 30 seconds and ultrasounding (40 kHz, 5℃) for 30 minutes. The samples were then placed at -20℃ for 30 minutes to precipitate proteins. After centrifugation at 13000g at 4℃ for 15 minutes, the supernatant was transferred to sample vials for LC-MS/MS analysis.

LC-MS analysis was conducted using a UHPLC-Q Exactive HF-X system (Thermo Scientific, USA). 2μL of sample was separated by HSS T3 column (100 mm × 2.1 mm i.d., 1.8 μm) and then entered into mass spectrometry detection. The mobile phase consisted of A = 0.1% formic acid in water:acetonitrile (95:5, v/v) and B = 0.1% formic acid in acetonitrile:isopropanol:water (47.5:47.5:5, v/v). We applied the following linear gradient: 0 - 24.5% B (0 - 3.5 min, 0.4 mL/min), 24.5 - 65% B (3.5 - 5 min, 0.4 mL/min), 65 - 100% B (5 - 5.5 min, 0.4 mL/min), 100% B (5.5 - 7.4 min, 0.4 - 0.6 mL/min), 100 - 51.5% B (7.4 - 7.6 min, 0.6 mL/min), 51.5 - 0% B (7.6 - 7.8 min, 0.6 - 0.5 mL/min), 0% B (7.8 - 9 min, 0.5 - 0.4 mL/min). The column temperature was maintained at 40 ℃ and injection volumes were 2 µL.

Data were collected with the following MS source settings: heater temperature, 425 ℃; capillary temperature, 325 ℃; sheath gas flow rate, 50 arb; aux gas flow rate, 13 arb; ion-spray voltage floating (ISVF), -3500V in negative mode and 3500V in positive mode, respectively; normalized collision energy, 20-40-60V rolling for MS/MS; mass range, 70-1050 m/z; resolution, 60000 (MS1), 7500 (MS/MS). Data acquisition was performed with the Data Dependent Acquisition (DDA) mode.

The raw data of LC/MS was preprocessed by Progenesis QI software (Waters Corporation，Milford, USA), and a three-dimensional data matrix in CSV format was exported. The information in this data matrix includes: sample information, metabolite name and mass spectral response intensity. Internal standard peaks, as well as any known false positive peaks (including noise, column bleed, and derivatized reagent peaks), were removed from the data matrix, deredundant and peak pooled. At the same time, the metabolites were searched and identified, and the main database was the HMDB (http://www.hmdb.ca/), Metlin (https://metlin.scripps.edu/) and Majorbio Database. The data were analyzed through the free online platform of majorbio choud platform (cloud.majorbio.com). Metabolic features detected at least 80% in any set of samples were retained. After filtering, minimum metabolite values were imputed for specific samples in which the metabolite levels fell below the lower limit of quantitation and each Metabolic features were normalized by sum, and the normalized data matrix was obtained. At the same time, variables with relative standard deviation (RSD) > 30% of QC samples were removed, and log10 logarithmization was performed to obtain the final data matrix for subsequent analysis.

The variance analysis was performed on the matrix file after data preprocessing. Principal component analysis (PCA) and orthogonal least partial squares discriminant analysis (OPLS-DA) were accomplished using the R package ropls (Version 1.6.2), and used 7-cycle interactive validation to evaluate the stability of the model. The selection of significantly different metabolites was determined according to the value of variable importance in the projection (VIP) (VIP>1) from the OPLS-DA model and the student’s t test (p<0.05).

**2.8. Norepinephrine (NE) level detection**

The fresh gastrocnemius muscle samples from the mice were rapidly homogenized. The supernatants were collected and used for the determination of NE concentrations using ELISA kit (Cusabio, Wuhan, China) according to the manufacturer’s instructions. NE level was detected by a luciferase reaction and read using a microplate reader (Envision 2104). The luminescence signal was correlated to NE concentrations according to a standard curve.

**2.9. Different cocktails of** **antibiotics (ABX) treatment**

ABX protocol containing ampicillin (100 U/mL, Sigma), neomycin (100 µg/mL, Sigma), gentamicin (170 µg/mL, Sigma), vancomycin (50 µg/mL, Sigma), and sucralose (4 mg/mL, Splenda, J&K), streptomycin (50 µg/mL, Sigma), metronidazole (100 µg/mL, Sigma), ciproﬂoxacin (125 µg/mL, Sigma) and ceftazidime (100 µg/mL, Sigma) in sterile drinking water for 4 weeks. Antibiotics were ad libitum and changed every 3 days.

1. **Original blots**


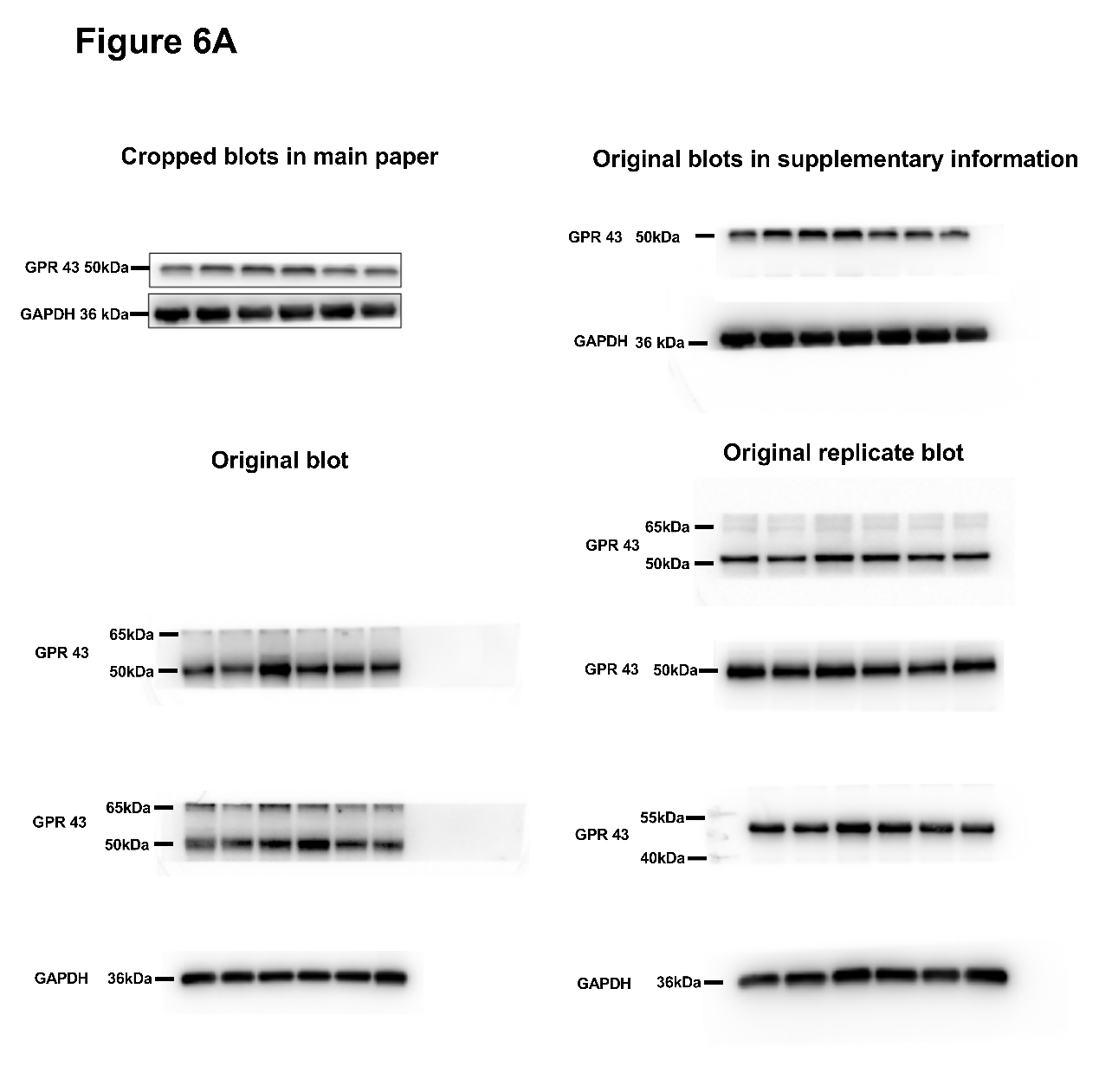


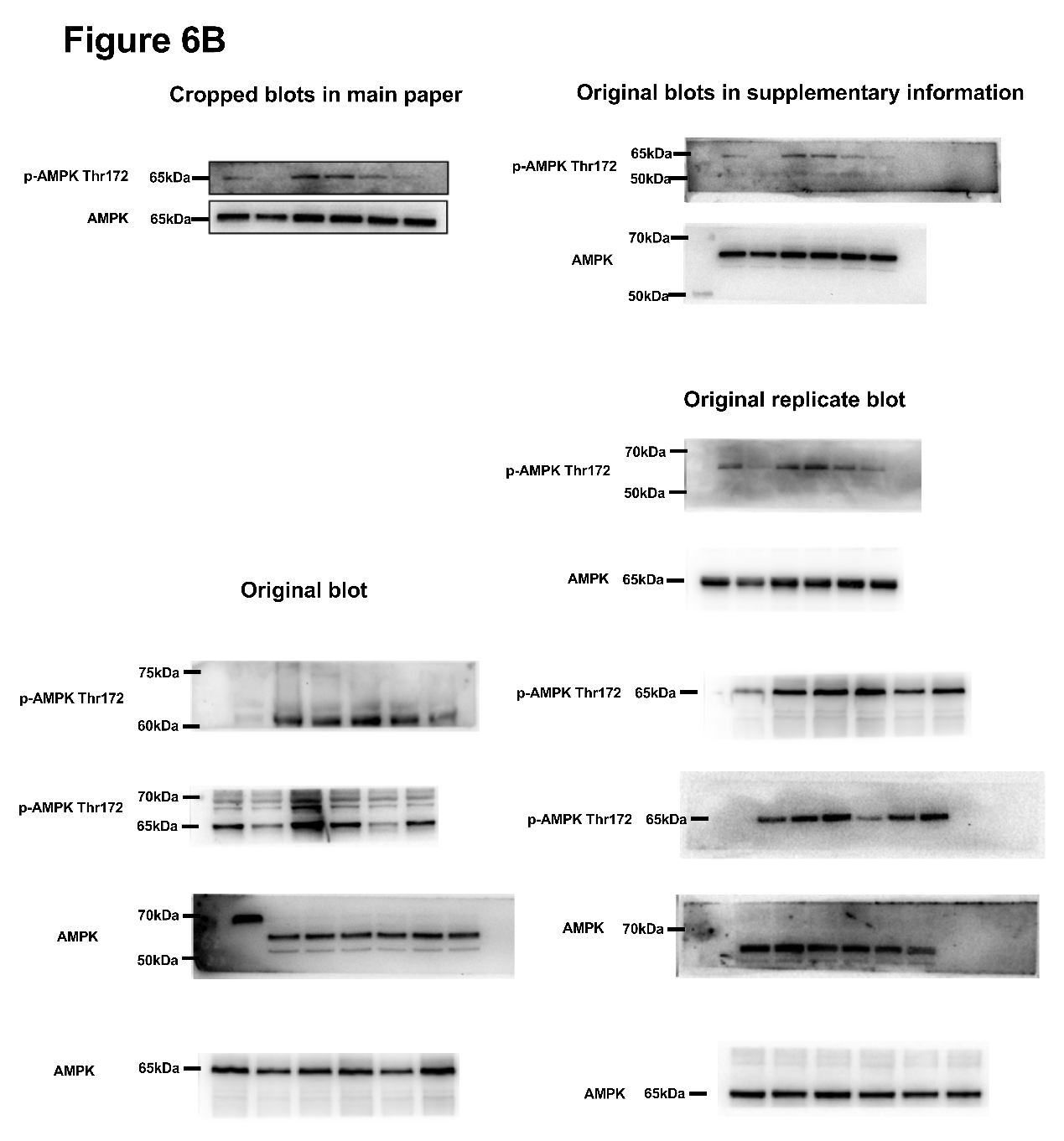


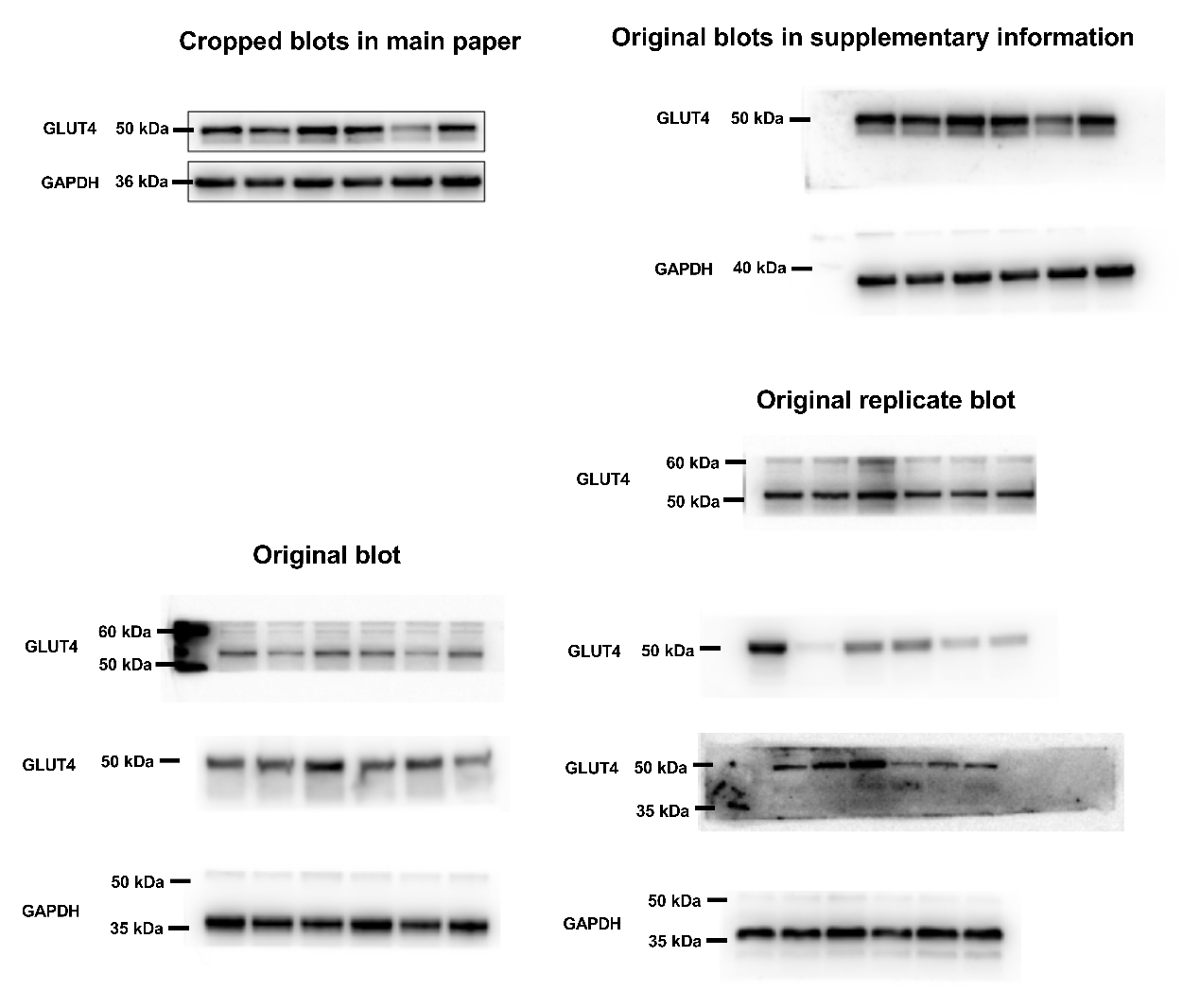


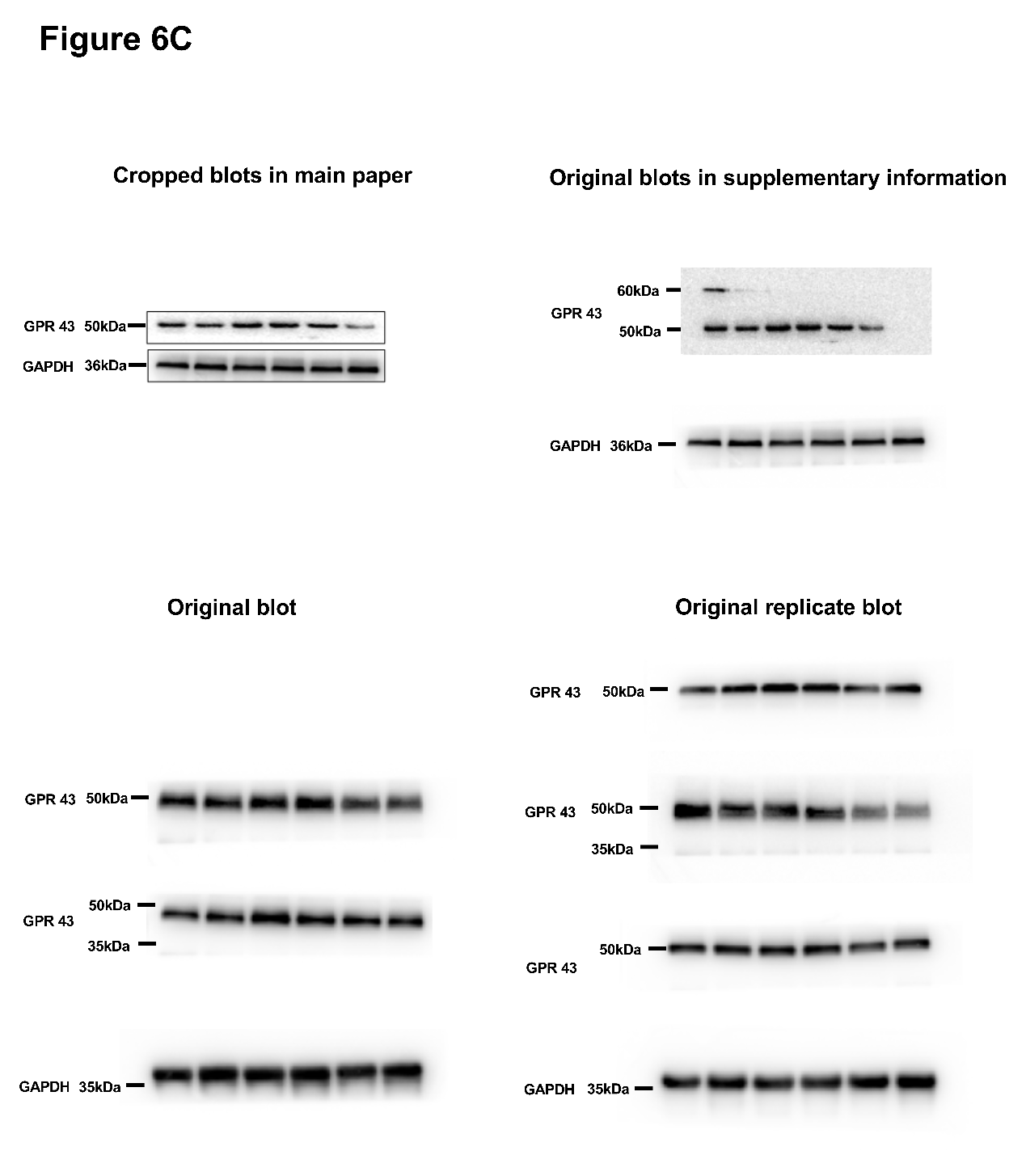


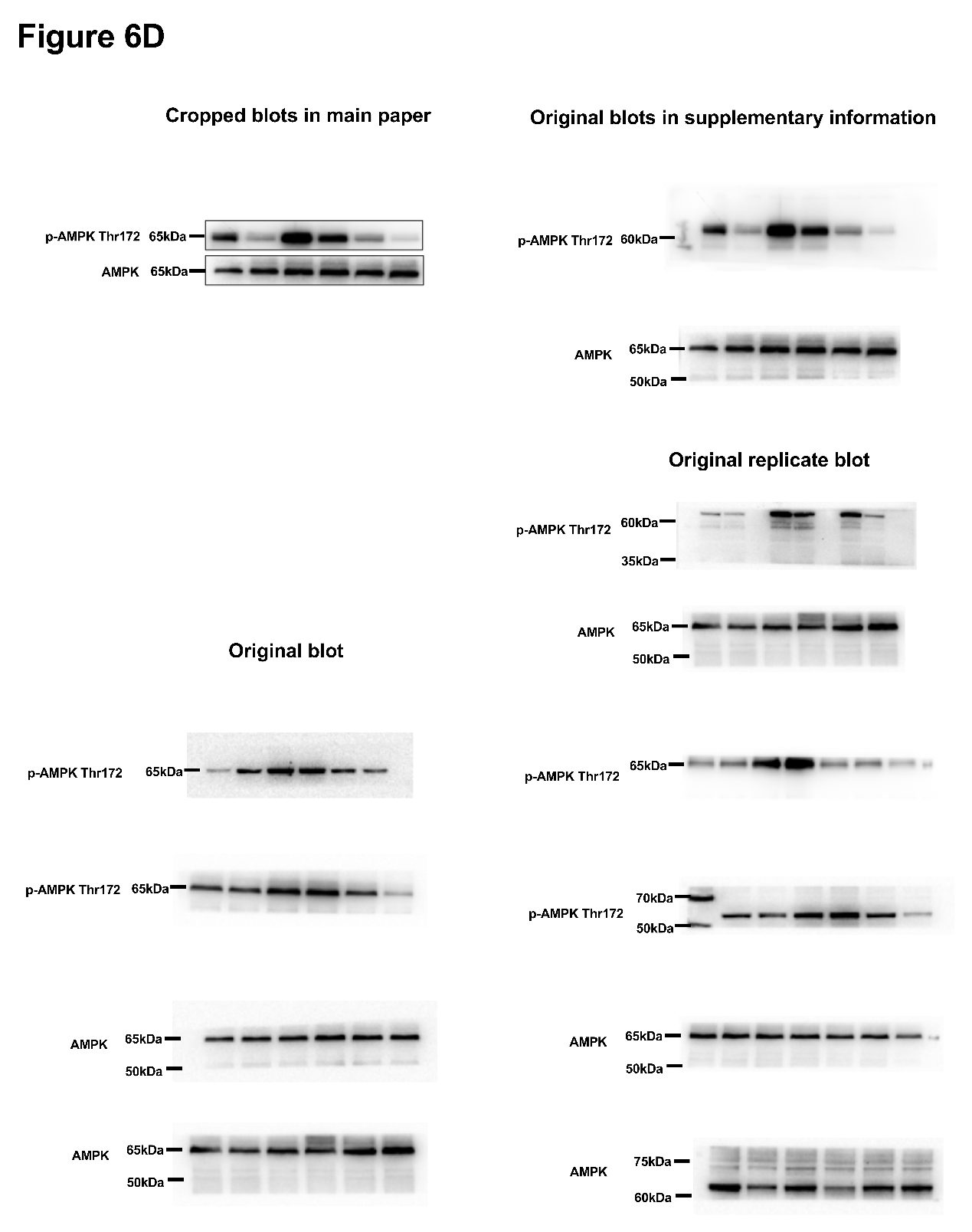


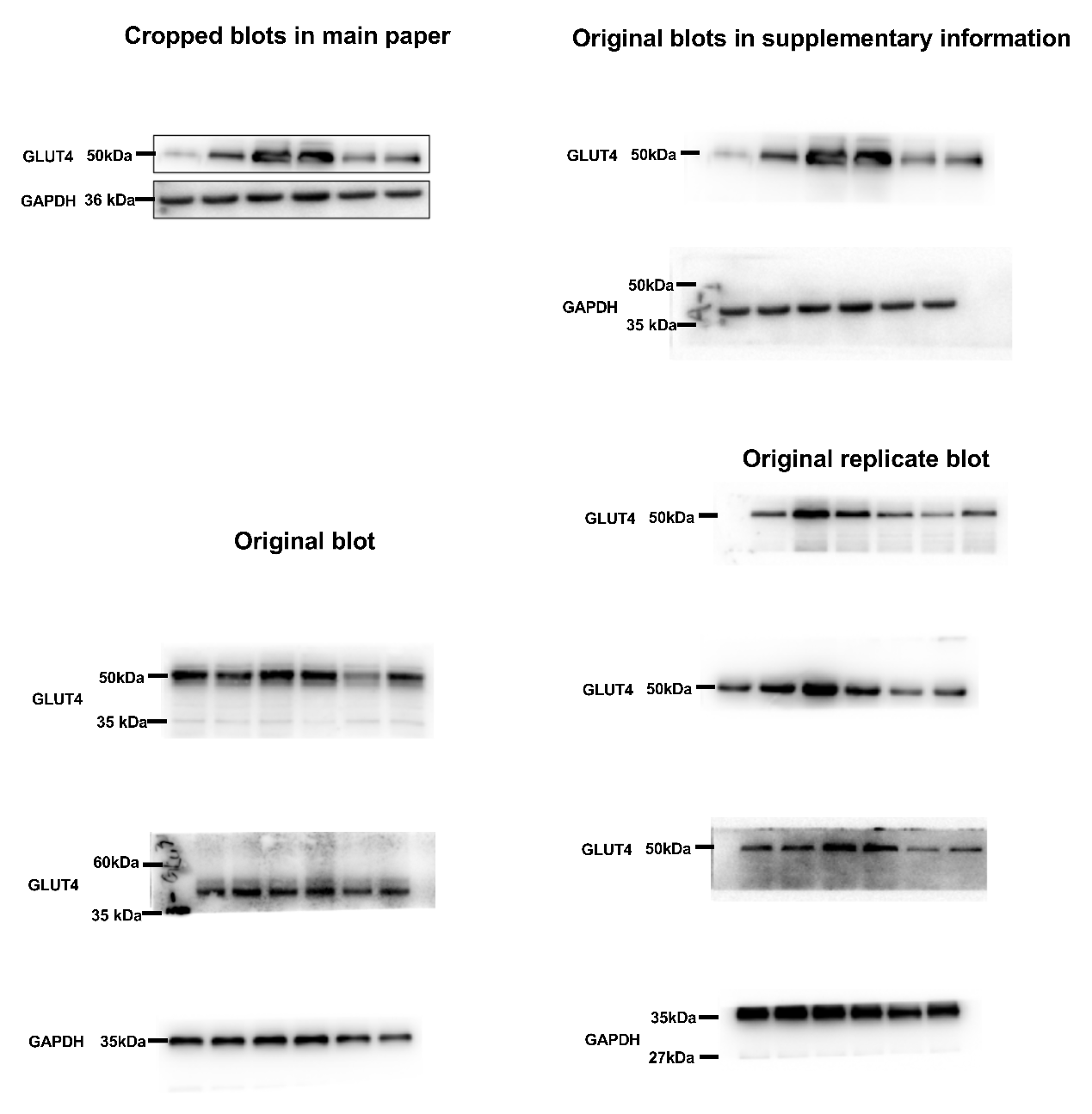


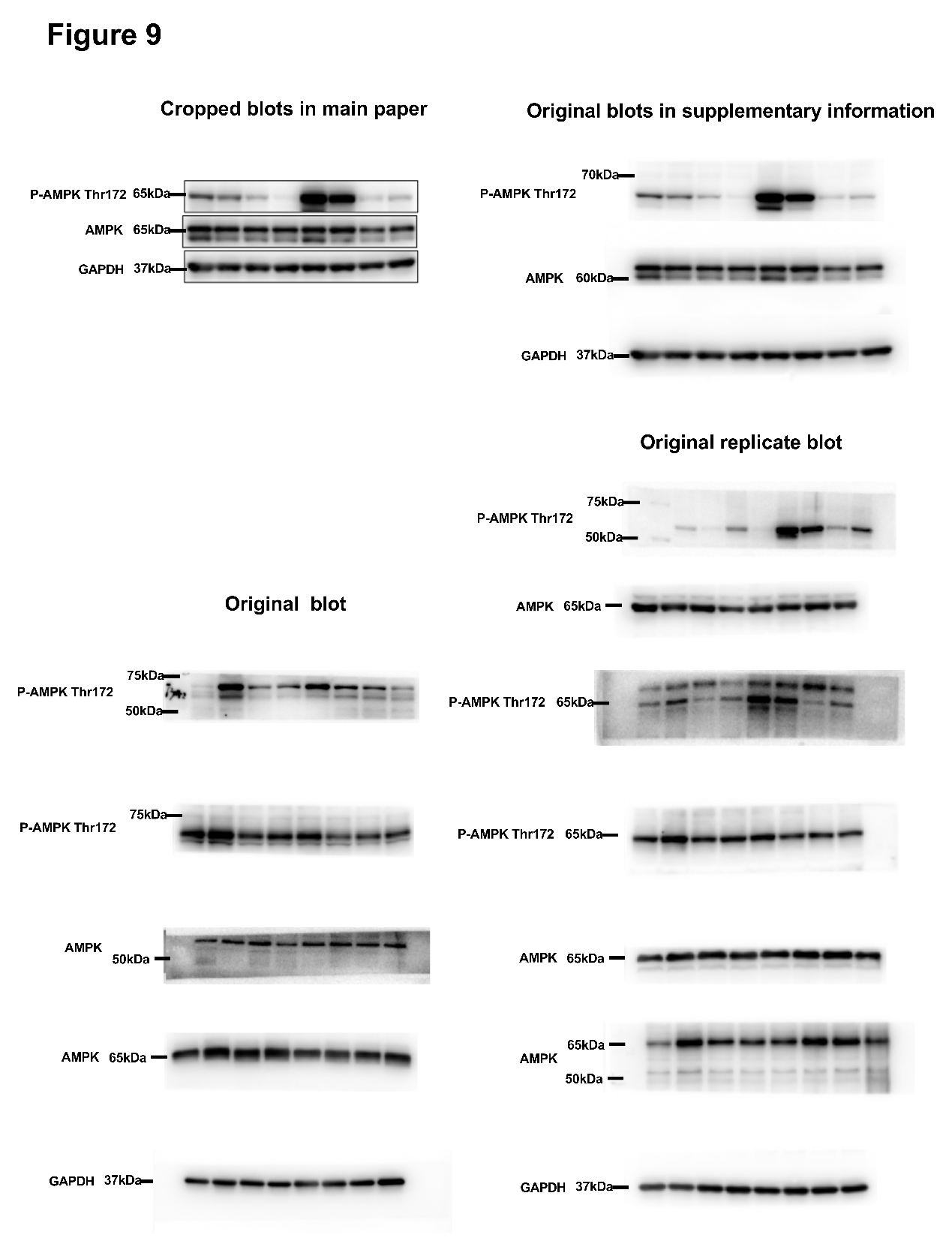


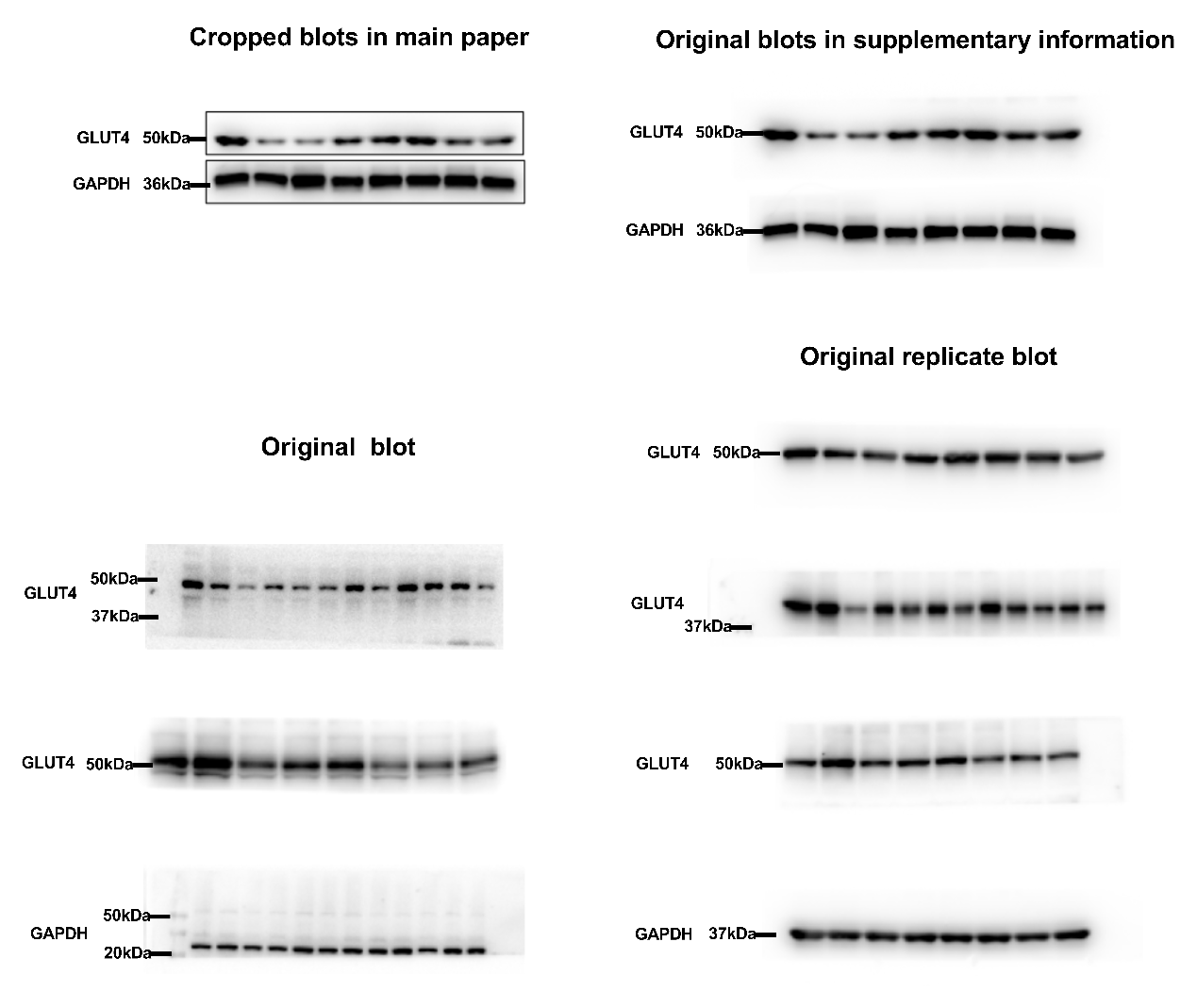

Supplement: Supplementary file 1 — Supplementary Information. [file 41598_2024_67220_MOESM1_ESM.docx]
